# Hippocampal spatio-predictive cognitive maps adaptively guide reward generalization

**DOI:** 10.1038/s41593-023-01283-x

**Published:** 2023-04-03

**Authors:** Mona M. Garvert, Tankred Saanum, Eric Schulz, Nicolas W. Schuck, Christian F. Doeller

**Affiliations:** 1 Max Planck Institute for Human Cognitive and Brain Sciences, Leipzig, Germany; 2 Max Planck Research Group NeuroCode, Max Planck Institute for Human Development, Berlin, Germany; 3 Max Planck UCL Centre for Computational Psychiatry and Ageing Research, Berlin, Germany; 4 Max Planck Institute for Biological Cybernetics, Tübingen, Germany; 5 Institute of Psychology, Universität Hamburg, Hamburg, Germany; 6 Kavli Institute for Systems Neuroscience, Centre for Neural Computation, The Egil and Pauline Braathen and Fred Kavli Centre for Cortical Microcircuits, Jebsen Centre for Alzheimer’s Disease NTNU, Trondheim, Norway; 7 Wilhelm Wundt Institute of Psychology, Leipzig University, Leipzig, Germany

**Keywords:** Decision, Hippocampus, Learning algorithms

## Abstract

The brain forms cognitive maps of relational knowledge—an organizing principle thought to underlie our ability to generalize and make inferences. However, how can a relevant map be selected in situations where a stimulus is embedded in multiple relational structures? Here, we find that both spatial and predictive cognitive maps influence generalization in a choice task, where spatial location determines reward magnitude. Mirroring behavior, the hippocampus not only builds a map of spatial relationships but also encodes the experienced transition structure. As the task progresses, participants’ choices become more influenced by spatial relationships, reflected in a strengthening of the spatial map and a weakening of the predictive map. This change is driven by orbitofrontal cortex, which represents the degree to which an outcome is consistent with the spatial rather than the predictive map and updates hippocampal representations accordingly. Taken together, this demonstrates how hippocampal cognitive maps are used and updated flexibly for inference.

## Abstract (web summary)

The authors find that the hippocampus creates a spatial and predictive cognitive map, and show how orbitofrontal and hippocampal computations select these maps adaptively to guide novel choices.

## Main

As humans, we live in complex, ever-changing environments that often require us to select appropriate behaviors in situations never faced before. Luckily, our environment is replete with statistical structure and our experiences are rarely isolated events^1^. This allows us to predict outcomes that were never experienced directly by generalizing information acquired about one state of the environment to related ones^2^. Indeed, humans and other animals generalize across spatially or perceptually similar stimuli^3–5^ as well as across stimuli forming associative structures such as those acquired in a sensory preconditioning task^6,7^. Generalization also occurs in reinforcement learning tasks where the same latent state determines the outcome associated with choosing different stimuli^8,9^.

For generalization to be possible, an appropriate neural representation of stimulus relationships is required. Many studies have shown that spatial relationships, such as distances between landmarks, are represented in a hippocampal cognitive map^10,11^, which enables flexible goal-directed behavior beyond simple stimulus-response learning^12^. More recently, it has been suggested that the same organizing principle might also underlie the representation of relationships between nonspatial states such as perceptual^13–17^ or temporal relationships between stimuli^18–21^, or associative links between objects^22–25^. Interestingly, cognitive maps even form incidentally and in the absence of conscious awareness^22^. This suggests that the hippocampus automatically extracts the embedding of a stimulus in relational structures^26,27^, even for stimulus features that are not directly task relevant^28^. In spatial navigation, stimuli can even be embedded in maps simultaneously, e.g., a policy-dependent predictive map reflecting the specific order in which stimuli are experienced during spatial navigation, as well as a policy-independent spatial (or Euclidean) map, that can be inferred from the subjective experience if one has prior knowledge about the topology of space.

If stimuli are part of several relational structures, this raises the question how the representation that is most beneficial for reward maximization and generalization can be selected^29^. One region implicated in this process is the orbitofrontal cortex (OFC), known to represent task states in situations where these are not directly observable^23,30^. Little is known, however, about how information in the OFC about the task-relevance of different maps relates to corresponding changes in the representation of cognitive maps in the hippocampus^31,32^.

Here, we combined virtual reality with computational modeling and functional magnetic resonance imaging (fMRI) to show that participants represent spatial as well as predictive stimulus relationships in hippocampal maps. The degree to which each dimension was represented neurally determined the degree to which it was used for generalization in a subsequent choice task, even though only the spatial location determined the magnitude of rewards. Notably, the neural representation of each map and its influence on choice changed over the course of the choice task through an OFC signal reflecting the relative accuracy of the predicted outcome based on the spatial as opposed to the predictive map. Together, our results provide a computational and neural mechanism for the representation and adaptive selection of hippocampal cognitive maps during choice.

## Results

### Participants used relational knowledge to generalize value

**Fig. 1:**
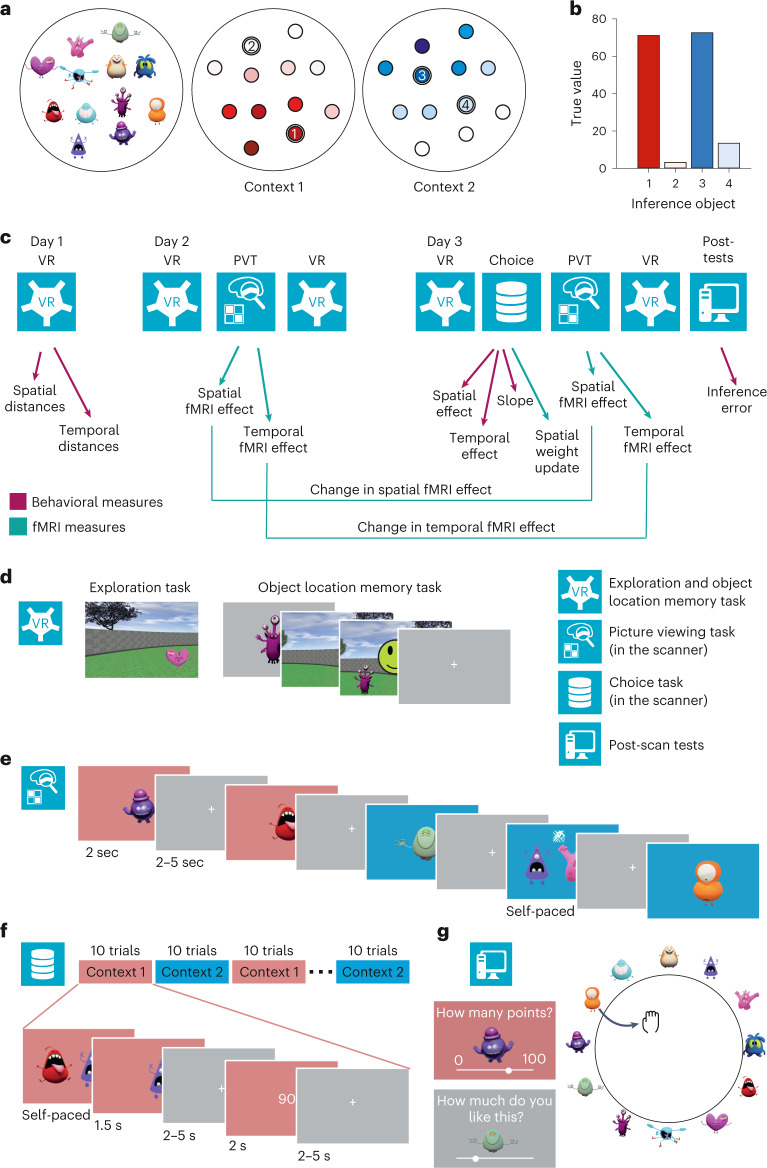
Experimental design. **a**, Spatial position of monsters during the navigation tasks and value distribution associated with the monsters in context 1 and 2 in the choice task. Darker colors indicate higher values (range: 0–100). Numbered circles indicate the location of inference stimuli that were never presented during the choice task. **b**, True values of the four inference stimuli. c, Tasks performed on the three subsequent days and glossary depicting the phase during which each behavioural and fMRI measure were computed. The study took place over three subsequent days and consisted of tasks where participants navigated around a virtual arena (VR, outside the scanner), a picture-viewing task (PVT, in the scanner), a choice task (in the scanner), as well as computer-based tests (outside the scanner). Arrows point to the measures extracted during each episode. Green arrows indicate behavioral measures, and purple arrows indicate fMRI measures. **d**, Exploration and object location memory tasks. In the exploration task (left), participants navigated around a virtual arena with button presses corresponding to forward, backward, right and left movements. Monsters appeared when they were approached, but were never all visible at the same time. In the object location memory task (right), participants were instructed to navigate to the position of a cued monster (each monster cued once in each block). Feedback indicated how far away the positioned stimulus was from the correct stimulus location. On day 1, participants performed between five and ten blocks (depending on performance) of the exploration and the object location memory task in alternation. On subsequent days, only one block of the object location memory task was performed before and after scanning without feedback. **e**, Picture-viewing task performed in the scanner. Participants were presented with monsters one after another. When two monsters appeared, participants were instructed to choose the monster that was closer in space to the preceding monster (map symbol) or the monster that was more similar in value to the preceding monster (coins symbol, day 3 only). On day 2, the background color was irrelevant for the task, on day 3 it indicated the context determining the stimulus values. **f**, Choice task performed in the scanner. Participants were instructed to maximize accumulated points by choosing the monster associated with a higher reward on each trial. Participants were told that the monsters had different values in two different contexts, and that the relevant context was signaled by the background color. The values associated with each monster in the two contexts were learned in alternation, with ten blocks of context 1 followed by ten blocks of context 2, and so forth. **g**, At the end of day 3, four post-tests were performed. Participants indicated for each monster how many points they would receive in each of the two contexts and how much they liked each monster. They were then asked to arrange the monsters in terms of their similarity in a circle in such a way that monsters that were considered similar were positioned near each other (Arena task 1). Lastly, participants were instructed to imagine a top-down view of the arena they had navigated around and to place the monsters in the corresponding location (Arena task 2).

To examine how humans use information about stimulus relationships for generalization and inference, 48 healthy human participants (mean age 26.8 ± 3.8 years, 20−34 years old, 27 male) took part in a 3-day experiment that involved learning to locate 12 monster stimuli in a virtual arena, followed by a choice task in which spatial knowledge could be used for predicting rewards (Fig. 1a).

**Fig. 2:**
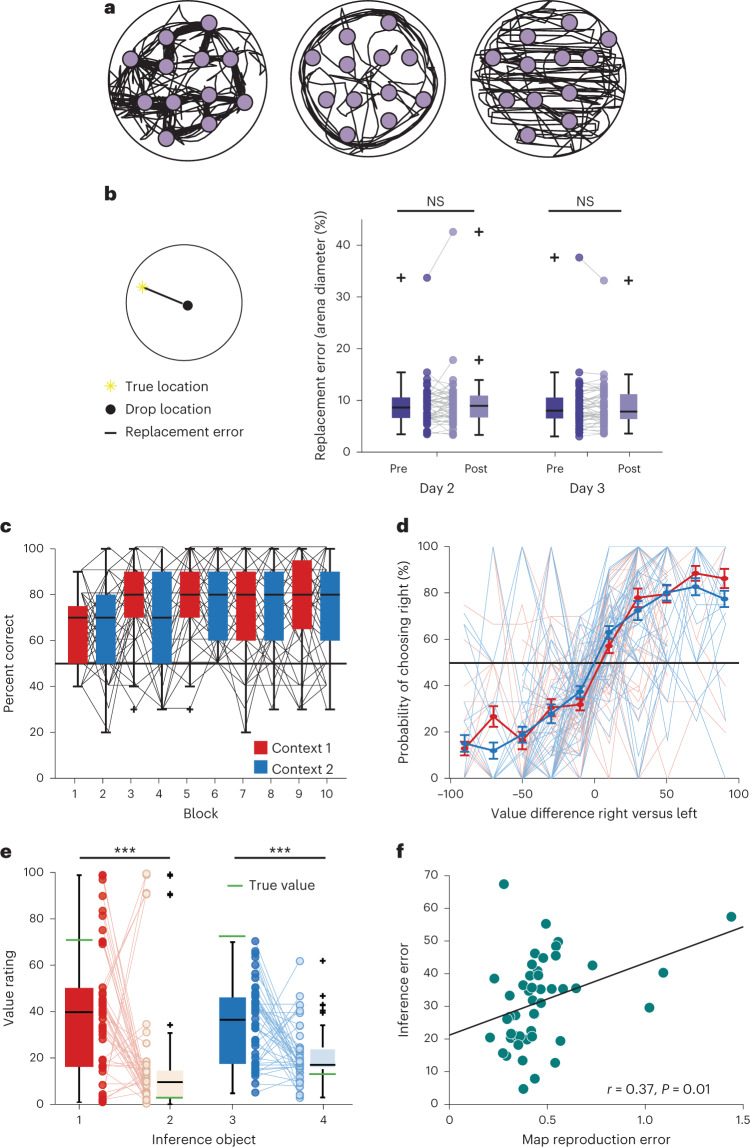
Behavioral results. **a**, Trajectories of three example participants during the exploration phase on day 1. Purple dots indicate the stimulus locations and black lines the participant trajectories. See all participants' trajectories in Extended Data Fig. 1. **b**, Replacement error for days 2 and 3, before (pre) and after (post) the scanning session. The replacement error was defined as the Euclidean distance between the true location and the drop location. The replacement error did not differ significantly between sessions (no significant main effect or interaction for session and condition (pre/post) in a two-way repeated measures ANOVA, *N* = 48, all *P* > 0.15), see stimulus positioning at the end of the learning phase on day 1 in Extended Data Fig. 2. **c**, Percentage correct of choices over the course of the choice task. Trials are divided into ten sub-blocks of ten trials each with a constant context (*N* = 48). **d**, Probability of choosing the right option as a function of the difference in value between the right and the left option, separately for each context. **e**, Value rating for the inference stimuli at the end of the study. Value ratings were significantly different between high- and low-value objects (*F*(1, 46) = 21.4, *P* < 0.0001), but not between contexts (*F*(1, 46) = 0.15, *P* = 0.70, two-way repeated measures ANOVA, *N* = 47). **f**, Correlation between the map reproduction error (root-mean-square error between the true *z*-scored spatial distances and the *z*-scored distances in the arena task) and the root-mean-square error for the inference ratings (Pearson’s *r* = 0.37, *P* = 0.01, CI (0.08, 0.59), *N* = 47). Data in **b**, **c** and **e** are plotted as group-level whisker-boxplots (center line, median; box, 25th to 75th percentiles; whiskers, most extreme datapoints the algorithm considers to be not outliers; crosses, outliers). Error bars in **d** denote s.e.m. Circles and transparent lines in **b**–**f** represent individual participant data, ***P < 0.001; NS, not significant. All statistical tests were two-sided. Source data

**Extended Data Fig. 1:**
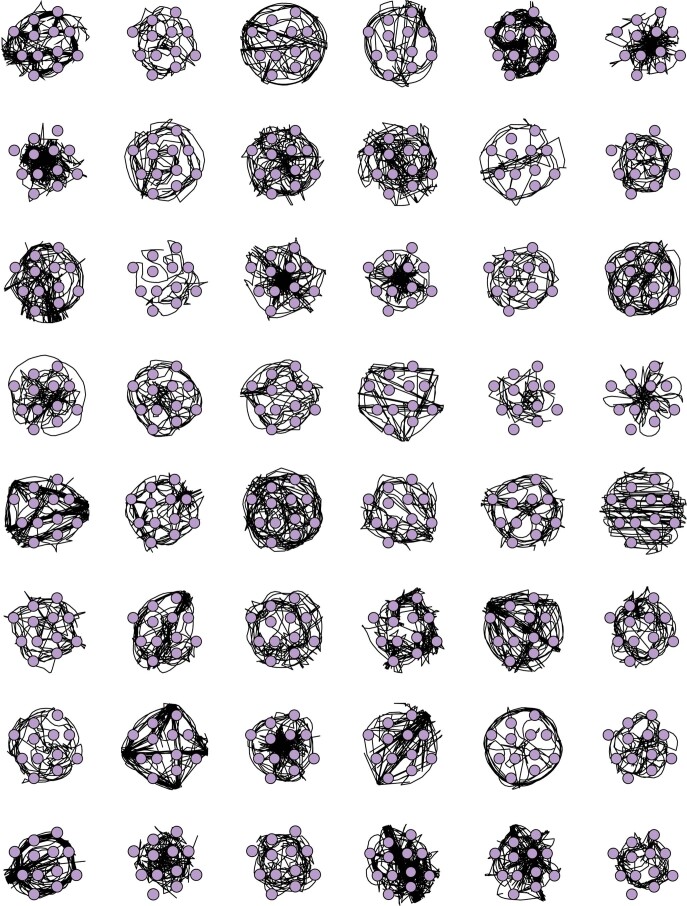
Exploration paths on day 1 in each individual. Each panel represents the exploration trajectories concatenated across exploration blocks on day 1 in one participant. Purple indicates the stimulus locations and black the participant’s trajectory.

On day 1, participants performed several exploration blocks in which they were instructed to remember the location of the stimuli while freely navigating in the arena (Fig. 1c,d). Stimuli became visible when they were approached, but were otherwise invisible. Exploration policies differed substantially between individuals (Fig. 2a and Extended Data Fig. 1). As a result, participants experienced different predictive relations between the monsters, which could also deviate from the spatial distances between stimuli. For example, some participants visited stimuli in a stereotyped order, whereas others navigated mostly around the border of the arena or systematically scanned the environment from top to bottom (Fig. 2a).

**Extended Data Fig. 2:**
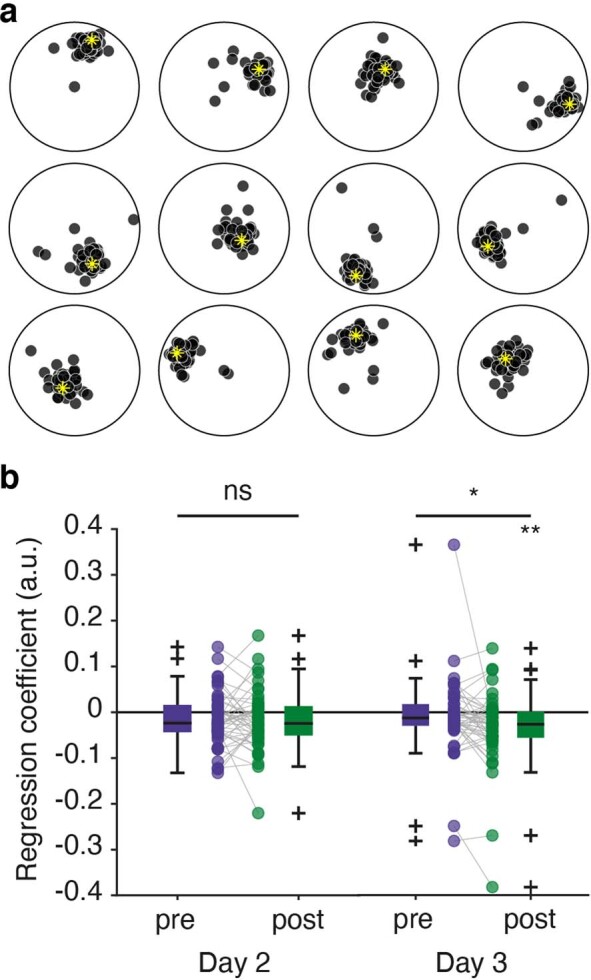
Stimulus positioning after learning. **a** Each panel displays the data for one stimulus. Yellow indicates the true stimulus position. Black indicates the drop location for each participant. The replacement error is defined as the Euclidean distance between the true location and the drop location. Visualized is the data from the last object location memory task block on day 1, that is at the end of learning. **b** Linear regression of values on replacement error. On day 2 as well as on day 3 before the choice task, there was no relationship between values participants learned to associate with each stimulus and replacement error (all p values > 0.05, N = 48). This is not surprising, since participants only learned the value associations on day 3. On day 3 after the choice task, the replacement error was smaller the higher the reported value of a stimulus (t(47) = -2.9, p = 0.005, N = 48, one-sample two-sided t-tests). The difference between value-dependent performance pre and post choice was also significant on day 3 (t(47) = 2.26, p = 0.03, N = 48, paired two-sided t-tests), but not on day 2 (t(47) = 0.27, p = 0.79, N = 48, paired two-sided t-tests). This suggests that participants’ memory expression was more accurate around valuable stimuli compared to less valuable ones after participants learned to associated stimuli with values. We used the average values that participants reported at the end of the study on day 3 as predictors. For inference stimuli, only the value experienced in the other context was considered. The central mark indicates the median, and the bottom and top edges of the box indicate the 25th and 75th percentiles, respectively. The whiskers extend to the most extreme data points not considered outliers, and the outliers are plotted individually using the ’+’ marker symbol.

After each exploration block, participants performed an object location memory task. Participants were teleported to a random location in the arena and instructed to navigate to the hidden location of a presented stimulus. Feedback indicated the magnitude of the replacement error (see Fig. 1c for a detailed description of all behavioral and fMRI measures). The session terminated when the replacement error averaged across all monsters in a block was below three virtual meters (vm; 3 vm corresponds to 10% of the arena’s diameter) and at least five and at most ten blocks had been completed. At the end of the learning phase, participants could position the stimuli in the correct location (Extended Data Fig. 2a). Before and after each imaging session on days 2 and 3, participants also performed one block of the object location memory task without feedback. The replacement error did not differ between sessions (Fig. 2b). In a spatial arena task at the end of the 3-day study, participants also accurately reproduced the stimulus arrangement when instructed to drag-and-drop stimuli imagining a top-down view on the spatial arena (Fig. 1g). Participants thus learned the spatial arrangement of the stimuli well.

In a choice task performed in the MRI scanner on day 3, participants were presented with two stimuli simultaneously and instructed to select the one that was associated with a higher reward (Fig. 1f). Participants were told that the reward magnitude was determined by the stimulus location in space (Fig. 1a). Participants could thus combine their knowledge about the stimulus relationships with previously experienced reward contingencies to infer the rewards of stimuli they had not yet experienced. To decorrelate spatial distance and reward relationships, we introduced two contexts with different reward distributions (Fig. 1a). Participants performed alternating choice blocks for each context, with the context signaled by the background color. Participants learned to perform the task rapidly (Fig. 2c) and their choices were a function of the difference in value between the stimuli presented on the left and the right on the screen in both contexts (context 1: *t*(47) = 10.0, *P* < 0.001, context 2: *t*(47) = 12.1, *P* < 0.001; Fig. 2d).

**Extended Data Fig. 3:**
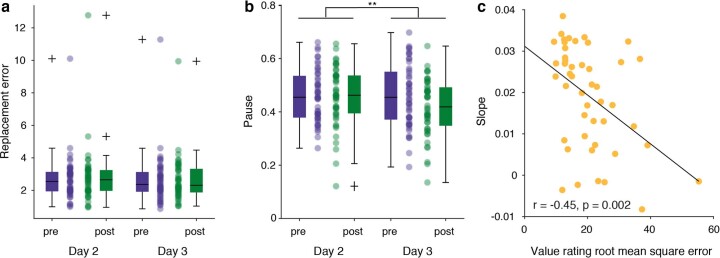
Effects of value learning on behavioral indicators of the map representation. **a** Replacement error in the object location memory task on days 2 and 3 before and after the scanning session. No significant change between sessions (main effect of session: F(1, 47) = 1.87, p = 0.18, main effect of pre/post: F(1, 47) = 0.86, p = 0.34, interaction: F(1, 47) = 0.37, p = 0.55, 2-way repeated measures ANOVA, N = 48). **b** Relative time spent in the same position during the object location memory task. Participants paused significantly less on day 3 after the value learning task (main effect of session: F(1, 47) = 7.77, p = 0.008, main effect of pre/post: F(1, 47) = 6.56, p = 0.01, interaction: F(1, 47) = 10.19, p = 0.003, 2-way repeated measures ANOVA, N = 48). **c** Relationship between root mean square error between true values and reported values at the end of the study and slope (Pearson’s r = -0.45, two-sided p = 0.002, CI: [-0.65, -0.18], N = 47), **p < 0.01. The central mark in **a** and **b** indicates the median, and the bottom and top edges of the box indicate the 25th and 75th percentiles, respectively. The whiskers extend to the most extreme data points not considered outliers, and the outliers are plotted individually using the ’+’ marker symbol.

To test whether participants could use their knowledge about the stimulus relationships to generalize, two stimuli per context were never presented during the choice task (‘inference stimuli’; Fig. 1a,b). At the end of the study, participants correctly inferred which of the two inference stimuli had a higher value in each context (repeated measures analysis of variance (ANOVA), *F*(1, 46) = 21.4, *P* < 0.001; Fig. 1g and Fig. 2e), demonstrating that participants exploited knowledge about stimulus relationships to infer unseen values. The error between the true inference values and the value ratings was larger in participants where the error between the true *z*-scored spatial distances and the *z*-scored distances in the arena task was larger (‘Map reproduction error’, *r* = 0.37, *P* = 0.01, robust regression *t*(45) = 2.31, *P* = 0.03; Fig. 2f). After the choice task, participants took less time for deliberation when navigating to a remembered stimulus location, perhaps pointing to a consolidation of the spatial map during value learning (Extended Data Fig. 3). Participants also positioned stimuli associated with high values closer to their true location (Extended Data Fig. 2). This suggests that participants’ memory expression was more accurate around valuable stimuli.

### Spatial and predictive relationships guide generalization

**Extended Data Fig. 4:**
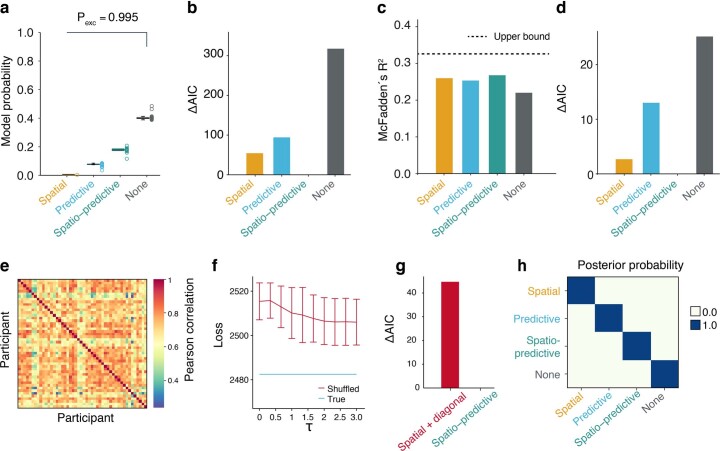
Full modeling results. **a** Each model’s probability of predicting individual participants’ value ratings for the experienced stimuli at the end of the study. Circles reflect each participant’s probability of being described best by each model. The winning model does not generalize about value (N = 48). **b** Model AIC differences for the choice task. **c** Models’ McFadden’s R^2 for the choice task, quantifying how likely a model is to produce the data relative to a random model, where a score of 1 means that the model is infinitely more likely to produce the data, and a score of 0 means the model is as likely as the random model. The dashed line represents the score of a model that uses the true value difference between options as a predictor on trials where participants had seen the values of both options. **d** Model AIC differences for predicting participants’ value ratings for the inference stimuli. **e** The pairwise correlation between all participants’ predictive kernels estimated with a learning rate of 0.4125, which gave the best fit for the predictive model. **f** Predicting reward generalization using participants’ own predictive kernel yields substantially better fits to their choice behavior (blue line) than predicting generalization using another randomly picked participant’s predictive kernel (red line, N = 48). Error bars are standard deviations of negative log-likelihood of 10 sampled random assignments. See Supplementary Note section 2 for procedure. **g** To verify that the predictive performance of the spatio-predictive model was not an artifact of the kernel composition procedure per se, we compared the spatio-predictive model against a model using a composition of a spatial kernel and the identity matrix. The results indicate that both the spatio-predictive model’s components captures something important about how participants generalized. **h** We performed a model recovery analysis for our computational models, using their own best-fitting hyper-parameters. We were able to recover each model’s behavior successfully. The entries in **h** show each model’s posterior probability of generating all simulated choice data sets, assuming a uniform prior. All models were by far the most likely to produce their own choice data. See Supplementary Note section 4. **a** are plotted as group-level whisker-boxplots (center line, median; box, 25th to 75th percentiles; whiskers, 1.5*interquartile range; crosses, outliers). Circles and transparent lines represent individual participant data.

Stimulus locations were learned during free exploration, which differed substantially between participants (Fig. 2a, Extended Data Fig. 1 and Extended Data Fig. 4e). Intelligent agents should keep track of both the spatial distance as well as the predictive relationships between stimuli experienced during navigation, since either feature may become relevant for generalization. We therefore reasoned that the brain may extract two relational maps: one reflecting spatial distances between stimuli and the other reflecting predictive relationships.

To test explicitly to what extent generalization was guided by the spatial or predictive maps—or a combination of both—we fitted Gaussian process (GP) models to participants’ choices (Online Methods). The GP predicts rewards for a new stimulus based on the rewards associated with all other stimuli, weighted by their similarity to the new stimulus. Since the similarity function determines how the GP generalizes, we can express hypotheses about what cognitive map participants use by pairing GPs with similarities implied by spatial or predictive maps.

Specifically, generalizing using a spatial cognitive map corresponds to pairing the GP with a similarity function that decays with Euclidean distance. Generalizing using a predictive cognitive map corresponds to pairing the GP with a similarity function that decays with predictive relations. We constructed these predictive similarities based on individual participants’ navigation runs from day 1: using their stimulus visitation history from the exploration phase, we computed each participants’ successor representation^33^, reflecting the expected number of visits of any stimulus ${s}^{{\prime} }$ given a starting stimulus *s*. This can be transformed into a probability that two stimuli are visited in direct succession (Online Methods). We then computed predictive similarities based on the diffusion distance^5^ implied by these transition probabilities.

Finally, kernel functions can be added or multiplied together to model function learning where generalization may be guided by a combination of multiple similarity functions^34,35^. As such, the hypothesis that both the spatial and predictive maps guide generalization together is captured in the spatio-predictive GP, which uses the additive composition of the spatial and the predictive similarities to generalize.

To test which map best explained how participants generalized rewards, we created three GP models that generalized based on either spatial, predictive or spatio-predictive relationships between monsters. Then, for each trial, we made each GP model predict the reward of both monsters, conditioning the GPs on all monster-reward pairs observed in the relevant context up to that point. We also compared these models with a ‘mean tracker’ model that assumes participants only learn about directly experienced stimulus-reward associations, without generalization (Online Methods).

To fit our models to participants’ choices, we entered the predicted difference in reward between the two presented monsters in a mixed-effect logistic regression model with random slopes per participant^36^, and determined the maximum likelihood hyperparameters using grid search. We then computed model frequency based on the leave-one-trial-out cross-validated log-likelihood for each model (Online Methods)^37^.

**Fig. 3:**
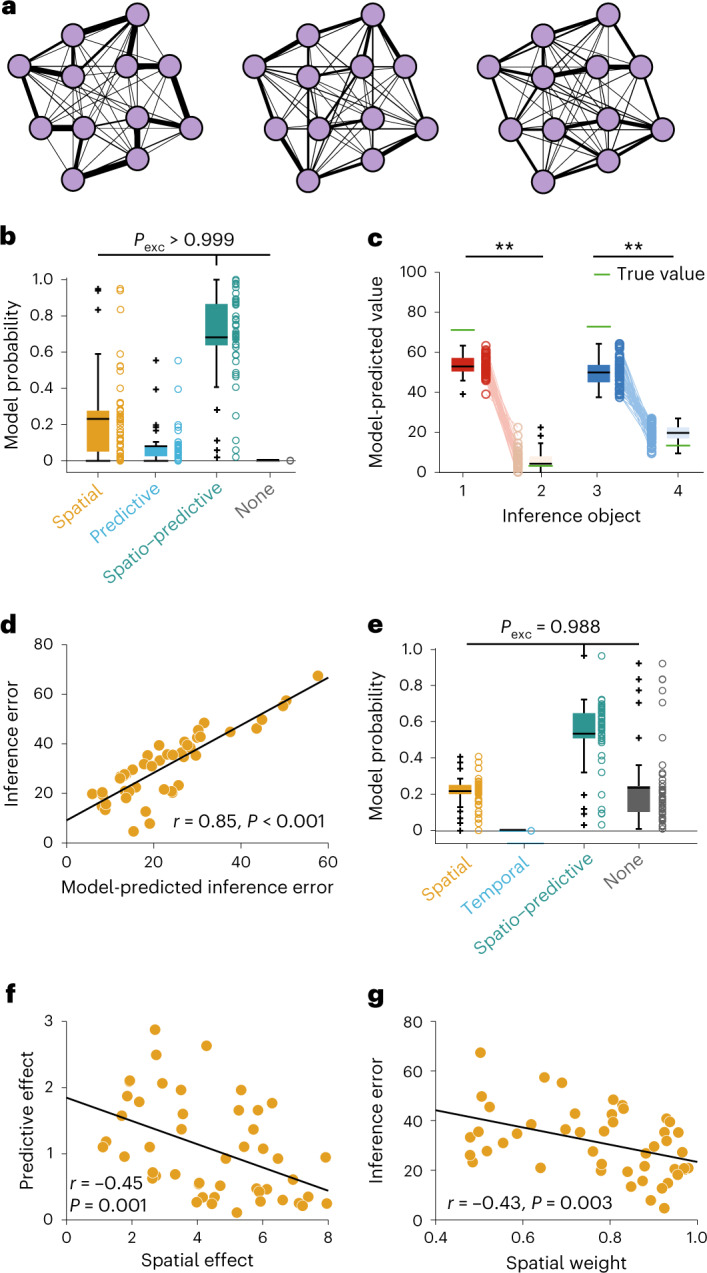
Modeling results suggest that participants generalized over spatial and predictive stimulus relationships. **a**, Graph representation of the three example exploration paths in Fig. 2a. **b**, Model comparison: model frequency represents how often a model fit to participantsʼ decisions in the choice task prevailed in the population. The winning model generalizes values according to a combination of spatial and predictive relationships between stimuli (*N* = 48). **c**, Inference performance as predicted by the model. Depicted are the inferred values for the inference stimuli in analogy to the participant ratings in Fig. 2e. Model-predicted values were significantly different between high-and low-value objects (*F*(1, 47) = 2,602.3, *P* < 0.0001) and between contexts (*F*(1, 47) = 81.2, *P* < 0.0001, two-way repeated measures ANOVA, *N* = 47). **d**, Relationship between inference error predicted by the model and actual inference error in participantsʼ value ratings (Pearson’s *r* = 0.85, *P* < 0.001, CI (0.75, 0.91), *N* = 48). **e**, Model comparison fit to the value ratings for the inference stimuli at the end of the study. The winning model generalizes values according to a combination of spatial and predictive relationships between stimuli (*N* = 47). **f**, Correlation between the spatial and predictive effects on choice behavior (Pearsonʼs *r* = − 0.45, *P* = 0.001, CI (−0.65, −0.20), *N* = 48). **g**, Correlation between the relative spatial weight as estimated by the model and inference error (Pearsonʼs *r* = − 0.43, *P* = 0.003, CI: (−0.64, −0.16), *N* = 48). Data in **b**, **c** and **e** are plotted as group-level whisker-boxplots (center line, median; box, 25th to 75th percentiles; whiskers, 1.5× interquartile range; crosses, outliers). Circles and transparent lines represent individual participant data. **P < 0.01. *P*_exc_, exceedance probability, which reflects the probability that a particular model is more frequent than all other models that were evaluated. All statistical tests were two-sided. Source data

The model generalizing based on the compositional, spatio-predictive similarities explained participants’ choices best (model frequency = 0.681, s.d. = 0.065, XP > 0.999; Fig. 3b and see Extended Data Fig. 4 for full modeling results). This model performed substantially better than the predictive model (model frequency = 0.08, s.d. = 0.038), the spatial model (model frequency = 0.23, s.d. = 0.059) and the mean tracker (model frequency = 0.005, s.d. = 0.01). The model also reproduced the difference in value rating for the high- and the low-inference stimuli (repeated measures ANOVA, *F*(1, 47) = 2,602.3, *P* < 0.001; Fig. 3c). Across participants, the root-mean-square error between true values and values predicted by the winning model was highly correlated with the root-mean-square error between the true values and the value ratings provided by participants (*r* = 0.85, *P* < 0.001, robust regression *t*(45) = 11.94, *P* < 0.0001; Fig. 3d).

Furthermore, participants’ value ratings for the inference stimuli at the end of the study were also predicted best by a spatio-predictive model (Fig. 2e). This demonstrates that behavior in two independent parts of the study, the choice task and the inference test, was influenced by both spatial and predictive knowledge about stimulus relationships. Notably, the value ratings for the stimuli whose values could be sampled directly were best predicted by the mean tracker model, rather than the spatio-predictive GP (Extended Data Fig. 4a). This suggests that participants evoked specific memories of stimulus-reward associations where possible, but relied on the spatio-predictive map when they needed to construct values of stimuli which were not experienced directly (Extended Data Fig. 4c).

We estimated effect sizes for the spatial and the predictive component as the participant-specific random effects in a model where the spatial and predictive regressors competed to explain variance in participants’ choices. Spatial weights were defined as the relative contribution of the spatial compared with the predictive regressor. Both the spatial and the predictive relationships had nonzero influence on choice behavior and the effect sizes were negatively correlated (Fig. 3f, *r* = − 0.45, *P* = 0.001, robust regression *t*(46) = −3.23, *P* = 0.002), suggesting that participants tended to rely predominantly on one of the two maps for guiding choice. Consistent with the fact that the spatial, but not the predictive relationships, were relevant for generalization, participants whose choices were driven more by the spatial relationships compared with the predictive ones performed better in the inference test (Fig. 3g, *r* = −0.43, *P* = 0.003, robust regression *t*(45) = −2.82, *P* = 0.007).

### Hippocampal spatial and predictive maps guide choice

**Extended Data Fig. 5:**
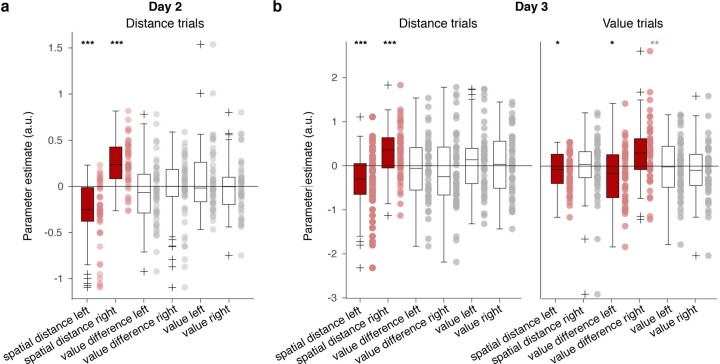
Logistic regression on participant behavior. We fit a multinomial logistic regression to participants’ choices in the scanner (coded 0 for occasions when participants chose left and 1 for occasions when participants chose right). Factors included were the spatial distance between the probe stimulus and the option on the left (1) and the right (2), the absolute difference in value between the probe stimulus and the option on the left (3) and on the right (4) as well as the value of the option on the left (5) and on the right (6). Models were fit separately for session 2 (**a**) and session 3 (**b**) and to distance and value trials (on session 3 only). **a** In line with the instructions, the spatial distance between the stimulus on the left and the probe stimulus influenced the probability of choosing left on distance trials on day 2 (factor 1, t(47) = -5.75, p < 0.0001, N = 48) and vice versa for the right side (factor 2, t(47) = 6.95, p < 0.0001, N = 48). No such relationship could be found for the difference in value between options and probe stimulus or for the reward magnitude itself (factor 3, t(47) = -1.38, p = 0.17; factor 4, t(47) = -0.70, p = 0.49; factor 5, t(47) = 1.55, p = 0.13, factor 6, t(47) = -0.23, p = 0.81, N = 48). **b** The spatial distance between the two stimuli and the probe stimulus influenced which stimulus a participant selected on distance trials on day 3 (distance left t(47) = -3.82, p = 0.0004, N = 48, distance right t(47) = 3.67, N = 48, p = 0.0006, all other p > 0.1, N = 48). **c** On value trials on day 3, the smaller the difference in value between the stimulus presented on the left side and the probe stimulus, the more likely that a participant would select this side (t(47) = -2.39, p = 0.02, N = 48) and vice versa for the right side (t(47) = 2.91, p = 0.006, N = 48). We also found a weak effect of the spatial distance between the stimulus on the left side and the probe stimulus (t(47) = -2.02, p = 0.049, N = 48), but no such effect for the right side (t(47) = -0.23, p = 0.82, N = 48). We found no effect for the reward magnitude associated with the two stimuli per se (both p > 0.1). The predictors that were significant without correcting for multiple comparisons are plotted in red. Statistical significance was inferred from the logistical regression. Data are plotted as group-level whisker-boxplots (center line, median; box, 25th to 75th percentiles; whiskers, most extreme datapoints the algorithm considers to be not outliers; crosses, outliers). Circles represent individual participant data. *p < 0.05, **p < 0.01, ***p < 0.001.

Our modeling results suggest that participants generalized values based on both the spatial and predictive relationships experienced during exploration. To investigate the neural representation of these relationships, we scanned participants before the choice task on day 2 and after the choice task on day 3 using fMRI. During these imaging sessions, stimuli were presented in random order on the two background colors (Fig. 2e). Once after each stimulus on each background color (that is, in 24 of 144 trials), participants were presented with two stimuli and instructed to report which one was either closer in space or more similar in value in the given context (on day 3 only) to the preceding stimulus. Participants performed this task well above chance (correct performance on day 2: 81 ± 10% (distance judgment); day 3: 78 ± 12% (distance judgment) and 68 ± 14% (value judgment), mean ± s.d., all *P* < 0.001) and choices were driven by spatial distances and value differences, respectively, and not by the absolute value associated with a monster (Extended Data Fig. 5).

**Extended Data Fig. 6:**
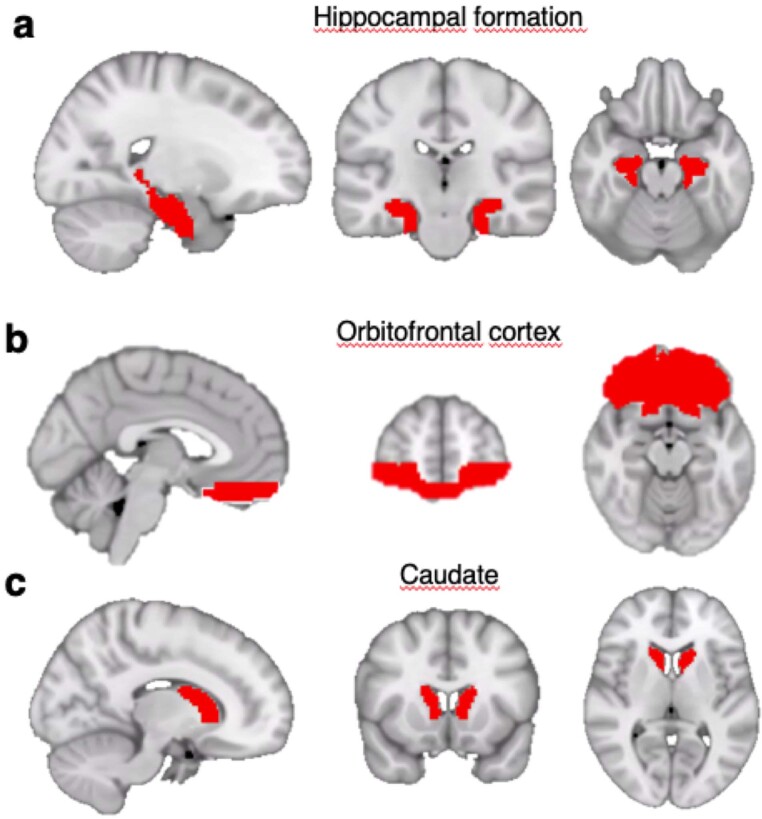
Anatomically defined regions of interest used for small-volume correction. **a** Mask of the hippocampal formation comprising bilateral hippocampus, entorhinal cortex and subiculum. **b** Mask of the orbitofrontal cortex. **c** Mask of the caudate.

We used fMRI adaptation^38,39^ to investigate the representational similarity of the 12 stimuli. This technique uses the amount of suppression or enhancement observed when two stimuli are presented in direct succession as a proxy for the similarity of the underlying neural representations. We hypothesized that, in regions encoding a cognitive map of the stimulus relationships, the size of the cross-stimulus adaptation effect should scale with spatial or predictive relations between stimuli. We tested for adaptation effects by including spatial and predictive distances as parametric modulators in the same general linear model (GLM). Based on previous work, we expected the hippocampal formation to be a candidate region for representing such cognitive maps^10,13,17,22,40^. All subsequent analyses are therefore reported at a cluster-defining threshold of *P* < 0.001, combined with peak-level family-wise error (FWE) small-volume correction (SVC) at *P* < 0.05. For the SVC procedure, we used a mask comprising hippocampus, entorhinal cortex, and subiculum (see mask used for small-volume correction in Extended Data Fig. 6a).

**Fig. 4:**
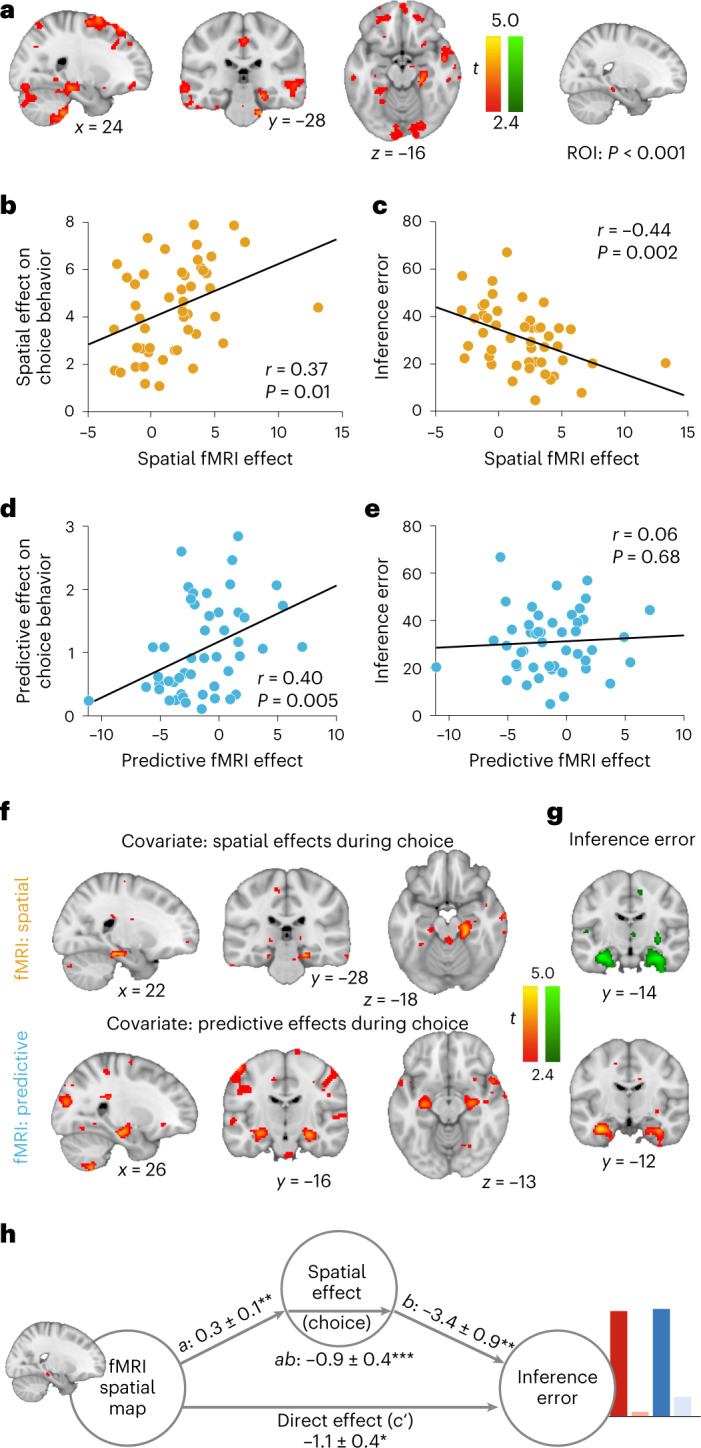
Spatial and predictive cognitive maps in the hippocampal formation are related to generalization and inference. **a**, Whole-brain analysis showing a cross-stimulus enhancement effect in the scanning session after the choice task (session 3) that scales with spatial distance. For illustration purposes, voxels thresholded at *P* < . 01 (uncorrected) are shown; only the right hippocampal cluster survives correction for multiple comparisons. **b**, Correlation between the spatial cross-stimulus enhancement effect extracted from the right hippocampal ROI depicted in **a** (thresholded at *P* < 0.001) and the spatial effects governing decisions in the choice task (Pearson’s *r* = 0.37, *P* = 0.01, CI (0.09, 0.59), *N* = 48). **c**, Correlation between the spatial cross-stimulus enhancement effect extracted from the right hippocampal ROI depicted in **a** and the root-mean-square error between ratings for the inference stimuli and their true value (Pearson’s *r* = −0.44, *P* = 0.002, CI ( − 0.65, − 0.18), *N* = 48). **d**, Correlation between predictive cross-stimulus enhancement effect extracted from the right hippocampal ROI depicted in **a** and the predictive effects governing decisions in the choice task (Pearson’s *r* = 0.40, *P* = 0.005, CI (0.13, 0.62), *N* = 48). **e**, Correlation between the predictive cross-stimulus enhancement effect extracted from the right hippocampal ROI depicted in **a** and the root-mean-square error between ratings for the inference stimuli and their true value (Pearson’s *r* = 0.06, *P* = 0.68, CI (−0.23, 0.34], *N* = 48). **f**, Whole-brain analysis where spatial effects (top) and predictive effects (bottom) describing generalization during choice are entered as second-level covariates for the spatial and predictive cross-stimulus enhancement effects. Both analyses reveal significant clusters in the hippocampal formation. **g**, Whole-brain analysis where the inference error is entered as second-level covariate for the spatial and predictive cross-stimulus enhancement effects. This analysis reveals a negative effect for the spatial map and a positive effect for the predictive map in the hippocampal formation. **h**, Mediation path diagram for inference error as predicted by the hippocampal map and spatial effects. Statistical inference was done using bootstrapping with 10,000 bootsamples. *a:* 0.3 ± 0.1, *P* = 0.01, *b:* − 3.4 ± 0.9, *P* = 0.004, *c':* − 1.1 ± 0.4, *P* = 0.02, *ab:* − 0.9 ± 0.4, *P* = 0.0004. **a**, **f** and **g** are thresholded at *P* < 0.01, uncorrected for visualization. *P < 0.05; **P < 0.01, ***P < 0.001. All statistical tests were two-sided. Source data

We found a significant cross-stimulus enhancement effect that scaled with spatial distance in session 3 (after the choice task) in the right hippocampal formation (Fig. 4a, peak *t*(47) = 3.86, *P* = 0.045, (24, −28, −16)). A cluster in the left hippocampal formation trended in the same direction (peak *t*(47) = 3.63, *P* = 0.08, (−12, −36 −6)). No voxels survived the conservative correction procedure for the predictive relations. One reason for this could be that different participants represented the spatial and predictive aspects to different degrees, with a stronger representation of the spatial map across the group as a whole. Indeed, in most participants (44 out of 48), the spatial component contributed more to generalization during choice than the predictive component (*t*(47) = 9.9, *P* < 0.001). We therefore investigated whether the strength of the neural representation predicted the degree to which an individual was influenced by either spatial or predictive relations in the choice task.

**Extended Data Fig. 7:**
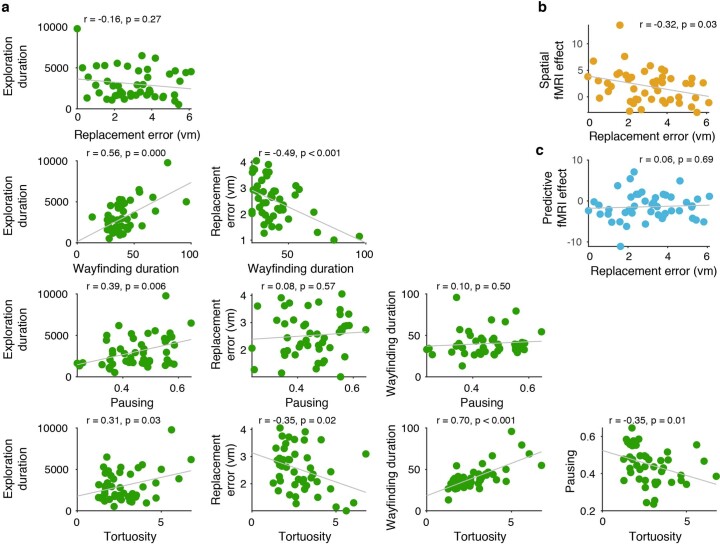
Relationship between navigational strategies and hippocampal spatial and predictive map representations. **a** Relationship between the following indices of navigational strategy across participants: **(1) Exploration duration**: Total duration of exploration across all exploration phases on day 1. **(2) Replacement error**: Measure of memory acuity. The replacement error was computed as the average Euclidean distance between the true stimulus location and the drop location in the object location memory task. We averaged this measure across sessions (days 2 and 3, before and after the scanning session). **(3) Wayfinding duration**: Average duration of an object location memory trial. **(4) Pausing**: Relative time spent in the same position during the object location memory task. Pausing was calculated by dividing the total number of time points spent not moving by the total time spent navigating in a trial in the object location memory task. **(5) Tortuosity**: Measure of the ability to directly navigate to a remembered stimulus location. Tortuosity was computed as the ratio of the length of the entire navigational path to the distance between its ends. This measure equals 1 for a straight line and is infinite for a circle. **b** Relationship between replacement error and the hippocampal spatial enhancement effect extracted from the ROI depicted in Fig. 4a across participants. **c** Relationship between replacement error and the hippocampal predictive enhancement effect extracted from the ROI depicted in Fig. 4a across participants. All r-values reflect Pearson correlation coefficients, p-values were computed for two-sided tests.

To test this, we extracted parameter estimates for the spatial and predictive maps from the region of interest (ROI) in the right hippocampal formation showing a cross-stimulus enhancement effect that scaled with spatial distance (masking threshold *P* < 0.001; Fig. 4a). A significant correlation with the spatial and predictive effects on choice behavior confirmed a relationship between the neural representation of the respective maps in this region and generalization behavior (spatial: *r* = 0.37, *P* = 0.01, robust regression: *t*(46) = 2.66, *P* = 0.01, predictive: *r* = 0.40, *P* = 0.005, robust regression: *t*(46) = 2.90, *P* = 0.006; Fig. 4b,d). We also found that the representation of the spatial, but not the predictive map in this ROI can be linked to performance in the later, independent inference test that depended on spatial knowledge (spatial: *r* = − 0.44, *P* = 0.002, robust regression *t*(45) = −3.1, *P* = 0.003; predictive: *r* = 0.06, *P* = 0.7, robust regression *t*(45) = 0.70, *P* = 0.49; Fig. 4c,e) as well as the replacement error in the object location memory task (Extended Data Fig. 7, spatial: *r* = −0.32, *P* = 0.03, predictive: *r* = 0.06, *P* = 0.69). Neither the formation of the spatial nor the predictive map was related to navigational strategies participants exhibited (Extended Data Fig. 7).

To investigate whether the relationship between spatial and predictive influences on behavior and neural map representation is specific to the hippocampus, we included spatial and predictive effects on choice behavior as covariates on the second level in the GLM that was used to identify spatial and predictive cross-stimulus enhancement effects. For both spatial and predictive maps, we found precisely localized clusters in the hippocampal formation, where the effects were larger the stronger the respective map’s influence on behavior (spatial: peak *t*(47) = 4.45, *P* = 0.009, [22, −28, −18], predictive: peak *t*(47) = 4.19, *P* = 0.02, [26, −20, −28], *t*(47) = 4.14, *P* = 0.02, [28, −14, −16] and peak *t*(47) = 3.91, *P* = 0.04, [−28, −16, −13]; Fig. 4f). Furthermore, the representation of the spatial map in the hippocampus was stronger and the representation of the predictive map was weaker in individuals who made smaller inference errors (spatial: peak *t*(47) = 5.08, *P* = 0.002, [32, −14, −25] and peak *t*(47) = 4.95, *P* = 0.002, [−32, −14, −22], predictive: peak *t*(47) = 4.53, *P* = 0.007, [−32, −12, −2]); Fig. 4g). This suggests that participants who represented the spatial map more strongly in the hippocampal formation also generalized more according to spatial distances in the choice task and performed better in the inference task, with the reverse pattern for the predictive relationships.

**Extended Data Fig. 8:**
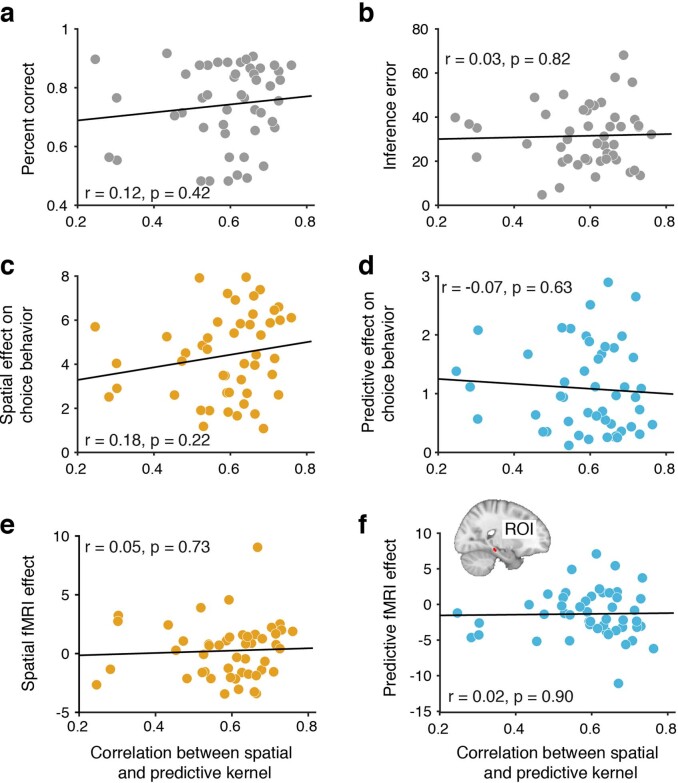
The correlation between spatial and the predictive kernels is not related to behavioral performance measures or hippocampal map representations. The correlation between the spatial and the predictive kernel is plotted against percent correct in the choice task (**a**) inference error (**b**), spatial effect on choice behavior (**c**), predictive effect on choice behavior (**d**) and fMRI cross-stimulus enhancement effect in the hippocampus for spatial (**e**) and predictive distances (**f**). Parameter estimates in **e** and **f** are extracted from the region of interest depicted in Fig. 4a. All r-values reflect Pearson correlation coefficients, p-values were computed for two-sided tests. None of the correlations reach significance (all p > 0.2).

**Extended Data Fig. 9:**
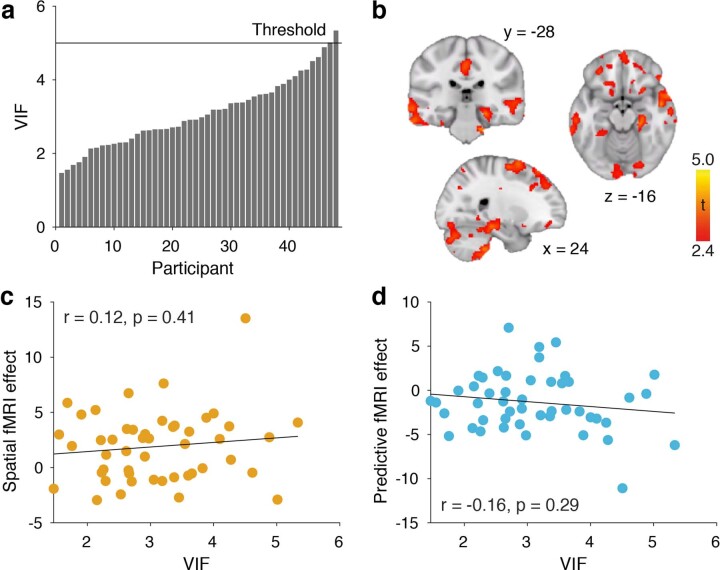
Relation of variance inflation factors (VIFs) with spatial and predictive effects. **a** Distribution of variance inflation factors (VIFs) for spatial and predictive regressors in the main GLM across participants. Participants are sorted according to the magnitude of the VIF. The threshold indicates a VIF greater than 5, which indicates potentially severe correlation between the spatial and the predictive regressors in the GLM. **b** Whole-brain analysis showing a cross-stimulus enhancement effect in the scanning session after the choice task (session 3) that scales with spatial distance. Participants with a VIF > 5 are excluded from this analysis. For illustration purposes, voxels thresholded at p > .01 (uncorrected) are shown; only the right hippocampal cluster survives correction for multiple comparisons (peak t(47) = 3.97; p = 0.035; [24; -28; -16]). **c** Correlation between VIF and spatial fMRI effect, r = 0.12, p = 0.41, CI = [-0.17, 0.39], N = 48. **d** Correlation between VIF and predictive fMRI effect, r = -0.16, p = 0.29, CI = [-0.42, 0.13], N = 48. All r-values reflect Pearson correlation coefficients, p-values were computed for two-sided tests.

To test whether the hippocampal spatial map formally mediated the impact of the neural representation on inference performance, we related the parameter estimates for the spatial map extracted from the right hippocampal ROI to both the spatial effects as estimated from behavior in the choice task as well as the inference performance using single-level mediation^41,42^. The path model jointly tests the relationship between the neural representation of the spatial map and the degree to which spatial relationships influenced generalization in the choice task (path *a*), the relationship between spatial weights in the choice task and inference performance (path *b*), and a formal mediation effect (path *ab*) that indicates that each explains a part of the inference performance effect while controlling for effects attributable to the other mediator. All three effects were significant (path *a*: 0.3 ± 0.1, *P* = 0.01, *b*: − 3.4 ± 0.9, *P* = 0.004, *c*’: − 1.1 ± 0.4, *P* = 0.02, *c*: − 1.9 ± 0.6, *P* < 0.001, *ab*: −0.9 ± 0.4, *P* = 0.0003; Fig. 4h). This confirms that the representation of a hippocampal cognitive map guides spatial generalization and inference during the choice task and the inference test. Furthermore, despite the fact that the spatial and the predictive kernel were correlated in most participants (average Pearson’s *r* = 0.58 ± 0.12), the neural effect as well as the degree to which behavior was influenced by either component could not be explained by a correlation between spatial and predictive kernels (Extended Data Fig. 8). Also the variance inflation factor as an index for the collinearity between GLM regressors for spatial and predictive kernels across participants was not related to the spatial or predictive fMRI effects (Extended Data Fig. 9).

### Representations of cognitive maps adapt to the task demands

**Fig. 5:**
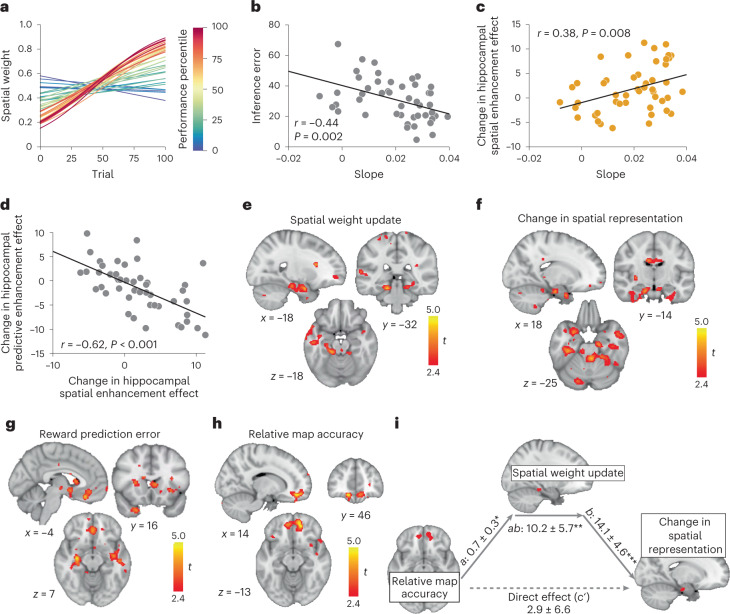
Hippocampal cognitive maps adapted to the task demands. **a**, Logistic functions for each participant fitted to how individual spatial weights changed over trials. Curves are colored according to a participant’s relative performance in the choice task. **b**, Correlation between the slopes of the estimated logistic function depicted in **a** and the inference error (Pearson’s *r* = −0.44, *P* = 0.002, CI (−0.64, −0.17), *N* = 47). **c**, Correlation between the slopes of the logistic function and the change in the hippocampal spatial enhancement effect extracted from the ROI depicted in Fig. 4a (Pearson’s *r* = 0.38, *P* = 0.008, CI (0.10, 0.60), *N* = 48). **d**, Correlation between the change in the hippocampal spatial and predictive enhancement effects. Both were extracted from the ROI depicted in Fig. 4a, (Pearson’s *r* = −0.62, *P* < 0.001, CI (− 0.77, −0.41), *N* = 48). **e**, Whole-brain analysis depicting the update in spatial weights at the time of feedback. **f**, Whole-brain analysis depicting voxels where the increase in the spatial cross-stimulus enhancement effect across participants correlates with the size of the hippocampal spatial weight update during the choice task as shown in **e**. **g**, Whole-brain analysis depicting voxels where the reward prediction error computed from a compositional map correlates with the size of the hippocampal spatial weight update during the choice task as shown in **e**. **h**, Whole-brain analysis depicting voxels where the relative accuracy based on the spatial versus the predictive map correlates with the size of the hippocampal spatial weight update during the choice task as shown in **e**. **i**, Mediation path diagram for the change in the hippocampal spatial cross-stimulus enhancement effect extracted from the ROI depicted in Fig. 4a as predicted by the OFC relative map accuracy signal and the hippocampal spatial weight update. Statistical inference was done using bootstrapping with 10,000 bootsamples. *a*: 0.7 ± 0.3, *P* = 0.02, *b*: 14.1 ± 4.6, *P* = 0.001, *c'*: 2.9 ± 6.6, *P* = 0.57, *ab*: 10.2 ± 5.7, *P* = 0.01. **e**–**h** are thresholded at *P* < 0.01, uncorrected for visualization. *P < 0.05; **P < 0.01; ***P < 0.001. All statistical tests were two-sided. Source data

We hypothesized that individuals adjust the degree to which they rely on one over the other dimension for guiding choice depending on the observed outcome contingencies. Indeed, a logistic function fitted to how individual weights changed over trials showed that, in most participants, the predictive component explained generalization behavior in the choice task better initially but, as the choice task progressed, spatial knowledge became more influential (Fig. 5a). The slope of this logistic function was steeper in participants who performed better in the choice task (Fig. 5a) as well as in the inference test (*r* = −0.44, *P* = 0.002, robust regression *t*(45) = 2.89, *P* = 0.006; Fig. 5b).

We reasoned that this might reflect changes in the representation of the neural map over the course of the choice task. If this is the case, then participants who showed a larger increase in the contribution of spatial knowledge on choices should also show a larger increase in the neural representation of the spatial map from day 2 (before the choice task) to day 3 (after the choice task). To test this, we extracted parameter estimates from the same ROI we used for the analyses in Fig. 4 for sessions 2 and 3 and correlated the difference with the slope of the logistic function. Indeed, participants whose behavior was characterized by increases in the reliance on the spatial map during choice also showed a larger increase in the neural representation of the spatial map (*r* = − 0.44, *P* = 0.002, robust regression *t*(45) = 2.58, *P* = 0.01; Fig. 5c). In the same region, the parameter estimate for the predictive map decreased significantly across participants (*t*(47) = − 2.1, *P* = 0.04) and the change in the spatial map representation was negatively correlated with the change in the predictive map representation (*r* = − 0.62, *P* < 0.001, robust regression *t*(45) = − 6.83, *P* < 0.0001; Fig. 5d), suggesting that, in participants where the spatial map representation became stronger the predictive map representation became weaker.

We reasoned that this change in representation might be driven by a neural signal reflecting the degree to which either map was task relevant. To test this hypothesis, we set up a GLM that included a parametric regressor that reflected the difference in the degree to which the spatial map influenced choice from one trial to the next (weight update signal). This identified a region in the left hippocampus (*t*(47) = 4.14, *P* = 0.02, [ −18, −32, −18]; Fig. 5e).

If this neural weight update signal led to an increase in the neural representation of the relevant map, then participants with stronger hippocampal weight updating signals should display a larger change in hippocampal representation of the spatial map from day 2 to day 3. To test where the spatial weight updating signal correlated with a change in the spatial map representation, we looked for changes in the spatial map representation from session 2 to session 3 across the whole brain, and included the parameter estimates extracted from the hippocampal ROI reflecting the spatial weight update as a covariate. This analysis revealed a significant positive effect in the left hippocampal formation (*P* = 0.018, *t*(47) = 4.21, [18, −14, −25]; Fig. 5g), suggesting that participants whose hippocampus tracked the spatial weight updates during the choice task also updated the representation of the spatial map in the hippocampus.

The changes in the composition of the hippocampal map likely reflect a representation learning process that was driven by the experienced reward contingencies in the choice task. We generated trial-wise reward prediction errors based on the compositional map and used this measure as a parametric modulator at feedback time. We reasoned that, if there is a relationship between the reward prediction error and the spatial updating signal, then fMRI activity should covary more with reward prediction error in participants whose hippocampal weight updating signal was stronger, and therefore included the spatial weight updating parameter estimate extracted from the hippocampal ROI as a covariate. Based on previous work in different species^43,44^, we hypothesized that the striatum and the OFC might play a particular role in tracking reward prediction errors and updating cognitive maps, respectively, and therefore used anatomically defined caudate and orbitofrontal cortex masks for small-volume correction (Extended Data Fig. 6b,c). Indeed, we observed significant clusters in the OFC (*t*(47) = 4.75, *P* = 0.02, [−4, 32, −20]), the striatum (right: peak *t*(47) = 3.57, *P* = 0.029, [12, 12, 16], left: peak *t*(47) = 3.63, *P* = 0.051, [−8, 16, 7]) as well as bilateral hippocampus (right peak *t*(47) = 5.06, *P* = 0.002, [30, − 16, − 30] and left peak *t*(47) = 4.00, *P* = 0.04, [−34, −16, −16], SVC using the hippocampal formation mask, Extended Data Fig. 6a), suggesting that the larger the hippocampal weight update, the more strongly these regions tracked reward prediction errors.

In addition to the reward prediction error itself, it may be useful for the brain to track how consistent the observed outcome is with the predictions made based on either of the two cognitive maps. This would allow the brain to adaptively adjust the cognitive map depending on task relevance. To test how predictable the observed outcome is based on the spatial or predictive maps, we calculated the trial-wise unsigned prediction errors for each outcome separately for the spatial and the predictive map. The difference between these two prediction errors indicates how much more expected an outcome was according to the spatial as compared with the predictive map. We then set up a GLM that modeled this difference at feedback time. Again, we reasoned that, if there is a relationship between this signal and the spatial updating signal, then participants whose hippocampal weight updating signal was stronger should also show more of such a relative map accuracy signal, and therefore included the parameter estimate extracted from the hippocampal ROI as a covariate. The only region where the relative map accuracy covaried with the hippocampal updating signal was the medial orbitofrontal cortex (*P* = 0.03, [14, 46, −13], FWE corrected on the cluster level; Fig. 5h).

This demonstrates that the more the hippocampus tracks the spatial weight update signal, the more the OFC signals both (1) how much the observed outcome diverges from the predicted reward based on the compositional map and (2) how consistent the observed outcome is with either of the two dimensions. This cannot be explained by a correlation between the two measures since the reward prediction errors are uncorrelated with the relative map accuracy regressors (average *r* = 0.017).

In line with the observation that the OFC adapts behavior by changing associative representations in other brain regions^45^, the orbitofrontal relative map accuracy signal may thus align task representation with observed outcomes. By signaling the degree to which either map is task relevant, spatial weights may be updated during the choice task, which in turn leads to an update of the spatial map representation itself. To test this assumption, we investigated whether the spatial weight update in the hippocampus formally mediated the relationship between the relative map accuracy signal in the OFC and the hippocampal changes in the spatial map representation. The fact that the OFC signal and the hippocampal spatial weight update was significant (path *a* = 0.7 ± 0.3, *P* = 0.02) is not surprising, since the ROI was identified based on voxels where the corresponding covariate explains some variance. However, the effect of the spatial weight updating signal on the change in representation remains significant if we control for the OFC signal (path *b* = 14.1 ± 4.6, *P* = 0.001). Furthermore, there is a relationship between the OFC signal and the change in hippocampal map representation (path *c* = 13.1 ± 6.6, *P* = 0.03), which can be fully accounted for by the hippocampal weight update (path *c*′ = 2.9 ± 6.6, *P* = 0.57, path *ab* = 10.2 ± 5.7, *P* = 0.01; Fig. 4h). Hence, participants with the largest OFC relative map accuracy signal at feedback time exhibited the largest updates in spatial weights in the hippocampus, which in turn related to a larger change in the neural representation of the spatial map. This suggests a role for OFC signal in adjusting the use of an appropriate map to the current task demands, and an associated behavioral change.

## Discussion

The hippocampal formation organizes relationships between events in cognitive maps, thought to be critical for generalization and inference. However, the neural and computational mechanisms underlying the ability to use cognitive maps for generalization remains unknown, especially in situations where stimuli are embedded in multiple relational structures. Here, we combined virtual reality, computational modeling and fMRI to demonstrate that the hippocampus extracts both spatial and predictive stimulus relationships from experience during navigation in a virtual arena. The strength of each neural representation was related to the degree to which it influenced behavior in an independent choice task. Notably, the OFC tracked the evidence that outcomes observed in the choice task were consistent with the predictions made by the spatial and the predictive cognitive map. This effect was more pronounced in those individuals where the hippocampus tracked the change in spatial weight on a trial-by-trial basis, perhaps suggesting a role of the OFC in adjusting the hippocampal map representation.

Because most individuals chose nonrandom behavioral policies for exploring the arena, stimulus relationships could be characterized both in terms of spatial distance as well as predictability. We found that the hippocampal formation extracted both types of relationships and represented those in clusters well known to represent distances to goals^46^, goal direction signals^47^ as well as associative distances between stimuli forming a nonspatial graph^22^. Notably, the degree to which either dimension was represented in this region determined the degree to which participant’s generalization behavior in a later choice task was influenced by the corresponding map. This links hippocampal representations of relational structures with generalization in decision-making. It also shows that this system deals efficiently with higher-dimensional relational structures and can combine information from multiple dimensions for guiding choice.

Our analyses are consistent with the formation of two distinct spatial and predictive maps of stimulus relationships. However, a more parsimonious account may be a single map where spatial information about the distances between monsters is distorted in an experience-dependent way. A combined map would lead to a similar fit to choice behavior as the composition of a spatial and a predictive map, making it difficult to make inferences about this based on the modeling results. However, the spatial map dominates behavior and is represented more strongly in the brain, whereas the predictive map seems to have a weaker, more modulatory, influence. The two dimensions are both located in the hippocampal formation and cannot be clearly separated anatomically. Furthermore, the change in the hippocampal spatial weight and the change in hippocampal predictive weight are negatively correlated, demonstrating the interdependence between the two dimensions. The change in weight we observe both behaviorally and neurally may thus reflect a refinement of the combined map driven by the choice task, where value information was consistent with the spatial, but not the predictive stimulus dimension.

Furthermore, participant choices became increasingly more influenced by spatial relational knowledge as the choice task progressed, suggesting that which map is used for guiding choice can be adaptively adjusted to the current task demands. This effect was related to an OFC evidence integration signal, indexing the difference in relative map accuracy for the spatial compared with the predictive map at feedback time. Participants whose OFC responded more strongly also showed a larger spatial weight updating signal in the hippocampus at feedback, which was, in turn, related to a stronger increase in the representation of the spatial map from before to after the choice task. This is consistent with the OFC tracking the evidence that the currently observable state of the world was driven by either of the two maps, and updating the degree to which either influences behavior accordingly.

Our findings are consistent with the proposed function of the OFC to represent state spaces, in particular in situations where the current state of the world is not readily observable and must be inferred^48^. The OFC is also typically involved in situations where participants need to adjust their behavior when outcome contingencies change^30^ or when memory responses require an arbitration between hippocampal and striatal inputs^49^. For example, reversal learning or outcome devaluation, where previously acquired cue-outcome and response-outcome associations need to be adapted, rely on an intact OFC^50^.

Importantly, our results also shed light on the interaction between OFC and the hippocampus. In line with previous observations indicating a relation between state representations in OFC and the hippocampus^31,51,52^, our results indicate that OFC might play an active role in learning state presentations in the hippocampus through experience^53^.

On the other hand, predictive information can be extracted directly from experience, whereas spatial information needs to be inferred from the experienced stimulus transitions. It is therefore also conceivable that predictive relations are represented earlier after learning, whereas the representation of spatial relations only emerges after a period of consolidation. The rehearsal of spatial knowledge associated with a successful performance in the choice task may also have contributed to a strengthening of the spatial representation. However, the links between OFC activity representing the evidence that an outcome is generated by either of the two maps, the spatial weight update signal in the hippocampus and the refinement of the hippocampal cognitive map suggest that the reward that is consistent with the spatial map plays an additional role in changing the neural representation and behavior. Unfortunately, the correlation between reward distribution and spatial distances makes it difficult to truly disentangle to what degree changes in the map representation are driven by experience with the spatial map or consolidation as opposed to reward feedback.

We found substantial interindividual differences in terms of the degree to which participants represented the spatial and predictive relationships a stimulus was embedded in neurally, and were influenced by those dimensions during choice. Indeed, in participants whose choices were influenced by the spatial or the predictive map, we found a cross-stimulus enhancement effect for spatial or predictive stimulus relationships, respectively. In participants whose choices were not influenced by those dimensions, on the other hand, the opposite was true: responses to a stimulus were suppressed if the preceding stimulus was close in space or time. Often, repetition suppression effects are more common than repetition enhancement effects in fMRI adaptation paradigms^38^. However, repetition enhancement effects have been reported both in single-cell recordings and in fMRI across sensory cortices^54,55^, in inferior frontal gyrus and anterior insula^24^ and in the hippocampus^56^. The neural mechanisms underlying repetition enhancement effects remain elusive^38^. In the visual cortex, repetition enhancement has been shown to result from disinhibition of inhibitory inputs^57^, but it is unclear whether similar mechanisms underlie enhancement effects in higher cognitive areas. Enhancement effects are often observed when stimuli are degraded, new or perceptually similar^58^. Also, behavioral relevance can influence the directionality of an fMRI adaptation effect. For example, while repetition suppression effects are typically observed in the hippocampus when a stimulus that is irrelevant for the task at hand is repeated, repetition enhancement effects can be observed in the same region when a stimulus is task relevant^56^. In our experiment, distances were highly relevant both for the choice task as well as the picture-viewing task that we used to measure stimulus representations. Alternatively, our results are also consistent with a differentiation of stimulus representations for stimuli that are close to each other in space and time. This is consistent with observations that multivoxel patterns in the hippocampal formation became more dissimilar for events that occurred close in time^40^, potentially reflecting a differentiation of stimulus representations that prevents interference^59,60^. It is conceivable that the decrease in stimulus similarity for nearby stimuli drive the effects we observe. Of course, if repetition suppression effects scale with the similarity of underlying neural representations^38^, then a decrease in similarity after learning about the monster locations would lead to the decrease in suppression—or increase in enhancement—that we see.

In conclusion, our results suggest that the hippocampus represents different dimensions of experienced relationships between stimuli in parallel. The degree to which each representation is used for guiding choice is governed by an OFC relative accuracy signal. The OFC is related to a spatial updating signal in the hippocampus, which is in turn related to a change in the representation of the spatial map. This provides a mechanistic insight into the way in which appropriate stimulus dimensions are selected for guiding decision-making in multidimensional environments.

## Methods

This study was approved by the ethics committee at the Medical Faculty at the University of Leipzig (221/18-ek) and complies with all relevant ethical regulations.

### Participants

A total of 52 neurologically and psychiatrically healthy participants took part in this study (mean age 26.8 ± 3.8 years, 20–34 years old, 27 male). All participants gave written informed consent before participation. Participants were recruited using the participant database of the Max Planck Institute for Human Cognitive and Brain Sciences. Due to a scanner defect, three participants could not complete the last day. One participant was excluded due to problems during the preprocessing. A total of 48 participants therefore entered the analyses. Two of those participants did not do the arena task at the end of the experiment, but their data were included in all other analyses.

### Experimental procedure

The experiment consisted of three parts performed on three subsequent days. On day 1, participants learned the stimulus distribution in a virtual arena. On day 2, we assessed the stimulus representation in the fMRI scanner. On day 3, participants performed a choice task to learn the rewards associated with each stimulus in the scanner. Afterwards, we again assessed the stimulus representations in the scanner. The sessions are described in more detail below. The exploration and object location memory task were coded using the Python-based virtual reality software package Vizard (v.4, WorldViz LLC). All other tasks were implemented in Matlab R2016a using Psychtoolbox v.3. Imaging data was preprocessed using fmriprep. Imaging and behavioral analyses were carried out with Matlab.

#### Day 1

Participants were first familiarized with the stimuli by being presented with the monsters one-by-one on the screen. They could click through the stimuli to proceed to the next one. Participants were then instructed that they would be asked to learn where each monster belongs in space, and that this knowledge would be important for collecting points in later sessions. Monsters were distributed in a circular arena with a virtual radius of 15 m (Fig. 1a). Which monster was presented in which location was randomized across participants. Five distinct trees were located behind the wall surrounding the arena, which functioned as landmarks. The location of the trees was randomized in such a way that one tree occurred at a random position in every 72° block in each participant. Tree locations were fixed across all experimental session.

Participants then learned the location of stimuli in space by navigating around a virtual arena (Fig. 1e) in multiple blocks. Each block consisted of an exploration phase and an object location memory task. In the exploration phase, participants navigated around the arena in any way they liked and for as long as they wanted. Whenever a participant approached a monster (that is, they entered a 3-m radius around the monster location), it became visible and slowly turned around its own axis. This means that participants never saw all monsters at the same time. After each exploration phase, participants performed an object location memory task. In this task, participants were cued with a monster and had to navigate to the corresponding location (Fig. 1f). Feedback indicated how close to the correct location a monster was positioned (<3 m, <5 m, <7 m, <9 m, >9 m). In each block, every monster had to be positioned once. The order was randomized. If performance reached a prespecified performance criterion of <3 m replacement error averaged across all monsters (corresponding to <10% error) in a block, the session terminated if a participant had completed at least five blocks. Participants performed a minimum number of five and a maximum number of ten blocks of this task to ensure that they had a good knowledge of the stimulus distribution.

#### Day 2

Before the scanning session, participants had another opportunity to explore the monster locations freely, followed by one more round of the object location memory task with feedback.

Subsequently, we assessed the monster representations in the scanner using a picture-viewing task. Here, participants were presented in the fMRI scanner with the monsters for 2 s in a random order on a red or a blue background, followed by an intertrial interval drawn from a truncated exponential function (2–5 s) with a mean of 3 s. Participants were instructed to view the images attentively. Occasionally (once after each monster on each background color), two monsters were presented simultaneously and participants had to indicate which of the two monsters was located closer in space to the monster they had seen immediately before the two monsters (self-paced). Participants received no feedback. The purpose of this task was to ensure that participants would always evoke the location a monster was embedded in during the stimulus presentations. Participants were instructed that the background color was irrelevant for performing the task. Each monster was presented six times on each background color (red, blue) per block, resulting in 144 stimulus presentations in each block. Participants completed three blocks of this task. Stimulus sequences were generated pseudorandomly using a genetic algorithm with the following constraints: Each stimulus in each context occurred the same number of times per block and no monster–monster transition was presented more than once.

After the scanning session, another round of the object location memory task was performed without feedback to assess participants’ memory for the monster locations.

#### Day 3

Before the scanning session, another round of the object location memory task was performed without feedback to assess participants’ memory for the monster locations.

In the scanner, participants then performed a choice task. Here, they were presented with pairs of monsters and instructed to select the monster that would lead to the highest reward. The reward distribution was related to the position of the monsters in space and the context as indicated by the background color (Fig. 1a). Participants were instructed that they would receive similar amounts of points for monsters located near each other in space. They learned the two value distributions in a blocked fashion, with ten trials of choices in context 1 alternating with ten trials of choices in context 2. Background colors and contexts were counterbalanced across participants. Value distributions were selected such that pairwise spatial distances and pairwise value differences across both contexts were not significantly correlated and that the overall value across all stimuli was similar across the two contexts.

Two stimuli in each context (‘inference stimuli’) could never be chosen during the choice task (Fig. 1a,b). These were later used to assess whether participants were able to combine information about rewards with information about the relationship between monsters to infer stimulus values that were never experienced directly. Critically, the value of one inference stimulus per context was high (71 and 72) and the value of the other inference stimuli was low (3 and 13).

Participants were presented with the two options until they indicated their selection by button press (self-paced). After a jittered intertrial interval, the outcome associated with the selection was presented for 2 s, followed by another jittered intertrial interval. Both intervals were again drawn from a truncated exponential function (between 2 and 5 s) with a mean of 3 s. Participants performed 100 trials of the choice task.

After the choice task, three blocks of the picture-viewing task were performed in the scanner. This time, the background color indicated the relevant context, and participants were instructed to think about each monster’s location in space and its associated value. Occasionally (once after each monster on each background color), two monsters were presented simultaneously and participants had to indicate which of the two monsters was located closer in space to the monster they had seen immediately before the two monsters or which monster had a more similar value. Which task was to be performed was indicated with a symbol presented above the two options. Correct answers were rewarded with €0.10. Stimulus sequences were the same as on day 2.

After the scanning session, another round of the object location memory task was performed without feedback to assess participants memory for the monster locations. This was followed by four brief tasks. (1) Participants had to indicate on a sliding scale from 0 to 100 how many points they would receive for each monster in each context, (2) participants rated on a scale from ‘not at all’ to ‘very much’ how much they liked each monster, (3) participants arranged monsters in an arena according to their similarity (Arena task 1) or (4) spatial location (Arena task 2). In each task, the order in which monsters were presented was randomized across participants.

### Reimbursement

Participants were paid a baseline fee of €9 per hour for the behavioral parts of the experiment and €10 per hour for the fMRI sessions. In addition, participants could earn a monetary bonus depending on performance. Points accumulated during the choice blocks were converted into money (100 points = €0.10). Furthermore, each correct choice during the picture-viewing task was rewarded with €0.10.

### Behavioral measures

- Spatial distance: measures the Euclidean distances between stimuli in the virtual arena. We obtained estimates of stimulus locations for every participant by performing path integration on their navigation runs.
- Predictive distance: measures the predictive distances between stimuli in the virtual arena (see Modeling for derivation of this measure).
- Replacement error: measures the Euclidean distance between the drop location and the true stimulus location in the object location memory task.
- Spatial effect (on choice behavior): measures the degree to which participants generalize along the spatial dimension in the choice task on day 3 according to the GP fit (Modeling). Per-participant measure.
- Predictive effect (on choice behavior): measures the degree to which participants generalize along the predictive dimension in the choice task on day 3 according to the GP fit (Modeling). Per-participant measure.
- Spatial weight: measures the relative size of the spatial effect versus the predictive effect in the choice task on day 3. A spatial weight of 1 means that the choices are only influenced by the spatial dimension. A spatial weight of 0 means that the choices are influenced only by the predictive dimension. Per-participant measure.
- Spatial weight update: measures the difference in spatial weight from one trial to the next during the choice task on day 3. Per-trial measure.
- Slope: slope of the logistic fit to spatial versus predictive weight during choice task on day 3. Per-participant measure.
- Reward prediction error: measures the reward prediction error generated based on a compositional map during the choice task on day 3. Used as a parametric regressor in a univariate fMRI analysis. Per-trial measure.
- Relative map accuracy: difference in unsigned prediction errors based on the predictive versus spatial map computed during the choice task on day 3. Used as a parametric regressor in a univariate fMRI analysis. Per-trial measure.
- Inference error: defined as the root-mean-square error between the true values of the inference stimuli and the error ratings provided by a participant in the post-scan test phase on day 3. Per-participant measure.
- Map reproduction error: measures the root-mean-square error between the true *z*-scored spatial distances between the monsters in the virtual arena and the *z*-scored distances between the monster positions in the arena task. We *z*-scored the distances to ensure that they had a comparable range.

### fMRI measures

- Spatial fMRI effect: measures the degree to which blood oxygen level-dependent (BOLD) activity in response to a stimulus during the picture-viewing task covaries with spatial distances to the preceding stimulus (repetition enhancement). Per-participant measure.
- Predictive fMRI effect: measures the degree to which BOLD activity in response to a stimulus during the picture-viewing task covaries with predictive distances to the preceding stimulus (repetition enhancement). Per-participant measure.
- Change in hippocampal spatial fMRI effect: difference in spatial fMRI effect from day 2 to day 3. Per-participant measure.
- Change in hippocampal predictive fMRI effect: difference in predictive fMRI effect from day 2 to day 3. Per-participant measure.
- Spatial weight update fMRI effect: measures the degree to which univariate BOLD signal during the choice task on day 3 covaries with the spatial weight update. Per-participant measure.
- Reward prediction error fMRI effect: measures the degree to which univariate brain activity covaries with the reward prediction error generated based on a compositional map during the choice task on day 3. Hippocampal spatial weight update is used as a covariate in this analysis. Per-participant measure.
- Relative map accuracy fMRI effect: measures the degree to which univariate brain activity covaries with the difference in absolute unsigned prediction errors for the spatial and predictive maps. Hippocampal spatial weight update is used as a covariate in this analysis. Per-participant measure.

### Modeling

We used GP regression to model reward learning and generalization in the choice task. GPs define probability distributions over functions $f \sim {{{\mathcal{N}}}}(m({{{{x}}}}),k({{{{x}}}},{{{{{x}}}}}^{{\prime} }))$, where *m*(*x*) is the mean function, giving the expected function values $\hat{{{{{y}}}}}$ at input points *x*, and $k({{{{x}}}},{{{{{x}}}}}^{{\prime} })$ the covariance function, or kernel, defining how similar any pair of input points, *x* and ${{{{{x}}}}}^{{\prime} }$, are. GPs can be updated to posterior distributions over functions by conditioning on a set of observed function outputs *y*. Here, the posterior mean function is given by

1 $${m}_{{{{\rm{post}}}}}({{{{x}}}})={{{{{k}}}}}^{{T}}{({{{{K}}}}+{\sigma }^{2})}^{-1}{{{{{y}}}}}^{{T}}$$

where *k* is the kernel matrix containing the covariance between training points and the evaluation points, *K* is the kernel matrix containing the covariance between all training points and *σ*^2^ is a diagonal variance matrix.

The hypothesis that generalization is guided by a spatial cognitive map corresponds to equipping a GP model with a Gaussian (or radial basis function) kernel, representing similarity as an exponentially decaying function of squared Euclidean distance. The Gaussian kernel defines similarity as follows:

2 $$k({{{{x}}}},{{{{{x}}}}}^{{\prime} })={\sigma }_{f}^{2}\exp \left(-\frac{\parallel {{{{x}}}}-{{{{{x}}}}}^{{\prime} }{\parallel }^{2}}{2{\lambda }^{2}}\right)$$

where ${\sigma }_{f}^{2}$ is a parameter controlling the degree to which the predictions differ from the mean, and *λ* is the lengthscale parameter, controlling how strongly input point similarity decays with distance. We obtained estimates of stimuli locations for every participant by performing path integration on their navigation runs. The path integration procedure consisted of tracking the changes in participants’ location from one time step to the next, adding a small amount of Gaussian noise (*σ* = 0.001) to the location estimates at each time point. A monster’s location was calculated as the average of the recorded positions that a participant was in whenever within a 3-m radius of that monster.

**Extended Data Fig. 10:**
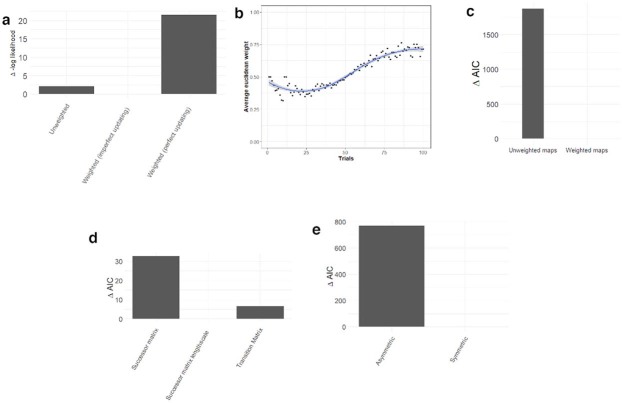
Supplementary modeling results. **a** We tested whether a map that adapts the weighting of the spatial and predictive kernels better captured participant behavior. We found evidence in the choice data that participants updated how they employed the maps based on its likelihood of generating the reward observations. See Supplementary Note section 5. **b** The trial-by-trial spatial weights, averaged over participants. Early in the choice task, the GP models have few observations to generalize from, and produce more similar predictions. Trial 1 & 2 and 11 & 12 have an equal weighting since the models have seen too few rewards to produce different predictions. The blue line and the shaded blue region correspond to mean and the 95% confidence interval estimated using a Loess regression model. **c** We fit a model using the estimated reward differences from the spatial and predictive maps as separate variables to predict participant choices. Crucially, these estimated reward differences were scaled by the weights we obtained on a trial by trial basis for each participant (average is depicted in **b**). This model outperformed the unweighted counterpart of this model by a substantial margin. **d** Model comparison of model learning the transition matrix by counting vs. models using the successor representation (SR) (learning rate = 0.001) to learn the transition matrix. See Supplementary Note section 6. **e** Model comparison of a model using asymmetric predictive relations and a model using symmetrical kernels to explain choice behavior (see Supplementary Note section 6).

To construct a kernel that corresponds to the hypothesis that predictive relations guided generalization, we started by computing a successor matrix *M* for every participant^33^. Each entry in the successor matrix ${{{{M}}}}(s,{s}^{{\prime} })$ (equation (3)) contains the expected discounted number of future visits of stimulus ${s}^{{\prime} }$, starting from a visit to stimulus *s*

3 $${{{{M}}}}(s,{s}^{{\prime} })={\mathbb{E}}\left[\mathop{\sum }\limits_{t=0}^{\infty }{\gamma }^{t}{\mathbb{I}}({s}_{t}={s}^{{\prime} })| {s}_{0}=s\right]$$

4 $$\hat{{{{{M}}}}}(s,:)\leftarrow \hat{{{{{M}}}}}(s,:)=\eta \left[{{{{\bf{1}}}}}_{s}+\gamma \hat{{{{{M}}}}}({s}^{{\prime} },:)-\hat{{{{{M}}}}}(s,:)\right]$$

where *γ* is the discount factor and ${\mathbb{I}}$ is the indicator function. The successor matrix can be approximated from a participant’s stimulus visitation history using a simple temporal-difference updating rule^61^ (equation (4)), where $\hat{{{{{M}}}}}(s,:)$ is the row corresponding to stimulus *s*, **1**_*s*_ is a vector of zeros except for the *s*th component which is a 1, and *η* is the learning rate. From *M* we computed the transition matrix *T* using the following equation (see Supplementary Note section 1 for derivation):

5 $${{{{T}}}}=\frac{{{{{{M}}}}}^{-1}-{{{{I}}}}}{-\gamma }$$

where *I* is the identity matrix. We enforced that *T* was symmetric by taking the pairwise maximum of the entries of its upper and lower triangles (Extended Data Fig. 10e). From *T*, which describes the relevant participant’s probabilities of walking directly from one stimulus to another, we computed the diffusion kernel^62^ *K*, embodying the hypothesis that predictive relations guide generalizations (equation (6)).

6 $${{{{K}}}}=\exp (-\lambda {{{{L}}}})$$

Here, $\exp$ is matrix exponentiation, *L* is the normalized graph Laplacian which equals *I* − *T* and *λ* is a lengthscale parameter analogous to that of the Gaussian kernel (equation (2)). Although we compute the transition matrix *T* by learning a successor matrix *M*, one could also estimate *T* by counting the number of times a participant transitioned directly between two stimuli, the transition probabilities being proportional to the count number. We found that computing the predictive kernel this way did not produce meaningful differences in model fit (see Extended Data Fig. 10d for an analysis involving asymmetric predictive relations).

To obtain the compositional kernel, we took the average of the Gaussian and the diffusion kernel^63^ and to implement the mean tracker, we used a GP model whose kernel was the identity matrix *I*. We assumed an equal weighting of the spatial and predictive kernel in the compositional kernel. Constructing the compositional kernel such that the weighting reflected the spatial and predictive GPs posterior probability of generating reward data did not improve fit to participant choices (see Extended Data Fig. 10a).

To obtain the various GP models’ estimates of stimuli’s rewards at any given trial in the choice task, we conditioned them on all previously observed stimuli’s rewards for the relevant context up to that point, and computed the posterior mean using equation (1). The differences in estimated rewards were used as single predictors of participant choices in a logistic mixed-effects model with a participant-specific random slope^36^, implemented in R using the lme4 package^64^. We optimized hyperparameters to minimize the log-likelihood of producing the choice data using a grid search. For the Gaussian kernel, we optimized the lengthscale *λ*, for the diffusion kernel we optimized the learning rate *η*, and set the discount rate parameter *γ* to 0.9 and the lengthscale *λ* to 1. For the compositional, spatio-predictive kernel, we optimized both the Gaussian kernel’s lengthscale and the learning rate. The variance in equation (1) was set 0.01 to improve numerical stability for matrix inversion. We first obtained each model’s hyperparameters that gave the best fit on the full choice dataset. Then, using these hyperparameters, we performed a leave-one-trial-out cross-validation (LOO-CV) procedure and obtained each model’s cross-validated log-likelihood of producing every choice in the dataset. We then computed the posterior model frequencies and exceedance probabilities^65^, as reported in Fig. 3b.

We used the same procedure for modeling participants’ value judgments. Here, we made the GP models predict the values of all stimuli, based on all reward observations the participants had made, respectively. The GPs were equipped with the best-fitting hyperparameters (Supplementary Note section 3) from the choice task. We then sought to predict participants’ value judgments for the different stimuli using the various value estimates as single predictors (plus an intercept) in separate linear mixed-effects models with a participant-specific random slope. We split the value judgments into two sets: one containing the value judgments of the inference stimuli, and another containing the value judgments of all monsters except the inference stimuli. Again, we performed LOO-CV to obtain model-specific log-likelihoods for all value judgments in the two datasets. Since the mean tracker could not generate predictions for the inference stimulus any different from its prior mean function (which was 0), we used the average of the mean tracker’s value predictions for the noninference stimuli as a baseline model. From the cross-validated log-likelihoods, we computed the corresponding sets of model frequencies and exceedance probabilities.

To compute the effects of the spatial and predictive components on each participant’s choice behavior, we fitted mixed-effects logistic regression models like the ones described above, using the estimated value differences generated by the spatial and predictive maps as individual predictors (using their respective best-fitting hyperparameters) in the same model. Since the two predictors were correlated, we created two such models, one where the spatial value difference was the main predictor, and the second predictor was the predictive minus the spatial predictor, and a second model where this relation was inverted^66^. We aggregated the unsigned mixed effects (random effects plus the fixed effects) across these two models for all participants, which left us with the effects for the two maps. To compute the spatial weights, we calculated how big the spatial effects were in proportion to the total effects (spatial plus predictive effects). The predictive weights were consequently 1 minus the spatial weights. To compute the slopes, we first obtained a weight for the spatial map for all trials and for all participants. We computed these weights by estimating two models similar to those used to estimate participant-specific effects, this time including an interaction term with trial number as well. To obtain trial-specific spatial weights for all participants, we estimated how likely the spatial by trial interaction predictor was at predicting each individual choice compared with the predictive by trial interaction predictor, aggregating over our two models. We found that weighing both maps’ predicted reward-difference by the estimated trial-by-trial weights improved fit to choice data (Extended Data Fig. 10c), providing confirmatory evidence that participants actually change weights over the course of the task. Moreover, we also found that the posterior probability of the spatial over the predictive model in generating reward observations for a particular participant at trial *t* was a significant predictor of the spatial weight estimated for that participant at trial *t* + 1, *t*(42.69) = 9.437, *P* < 0.001, establishing a connection between how well the spatial map explains the reward data at *t*, and how much participants rely on the spatial map for generalization on the subsequent trial. We also observed that the time-series of trial-by-trial spatial weights (averaged over participants) resembled a logistic function, going from lower spatial weights early in the task, to higher weights later (Extended Data Fig. 10b). We then fitted logistic slopes to each participant’s spatial weight time-series, predicting spatial weights for single participants from trial number, using logistic regression.

### MRI data acquisition and preprocessing

Visual stimuli were projected onto a screen via a computer monitor. Participants indicated their choice using an MRI-compatible button box.

MRI data were acquired using a 32-channel headcoil on a 3 T Siemens Magnetom SkyraFit system (Siemens). fMRI scans were acquired in axial orientation using T2*-weighted gradient-echo echo-planar imaging (GE-EPI) with multiband acceleration, sensitive to BOLD contrast^67,68^. Echo-planar imaging (EPI) with sampling after multiband excitation achieves temporal resolution in the subsecond regime while maintaining a good slice coverage and spatial resolution^67,68^. We collected 60 transverse slices of 2 mm thickness with an inplane resolution of 2 × 2 mm, a multiband acceleration factor of three, a repetition time of 2 s, and an echo time of 23.6 ms. Slices were tilted by 90° relative to the rostrocaudal axis. The first five volumes of each block were discarded to allow for scanner equilibration. Furthermore, a T1-weighted anatomical scan with 1 × 1 × 1 mm resolution was acquired. In addition, a whole-brain field map with dual echo time images (TE1 = 5.92 ms, TE2 = 8.38 ms, resolution 2 × 2 × 2.26 mm) was obtained to measure and later correct for geometric distortions due to susceptibility-induced field inhomogeneities.

#### Anatomical data preprocessing

Results included in this manuscript come from preprocessing performed using *fMRIPrep* v.1.4.0 (refs. ^69^) (RRID:SCR_016216), which is based on *Nipype* v.1.2.0 (refs. ^70,71^) (RRID:SCR_002502).

A total of two T1-weighted (T1w) images were found within the input BIDS dataset. All were corrected for intensity nonuniformity (INU) with N4BiasFieldCorrection^72^, distributed with ANTs v.2.2.0 (ref. ^73^). The T1w reference was then skull-stripped with a Nipype implementation of the antsBrainExtraction.sh workflow (from ANTs), using OASIS30ANTs as target template. Brain tissue segmentation of cerebrospinal fluid (CSF), white matter (WM) and gray matter (GM) was performed on the brain-extracted T1w using fast^74^. A T1w reference map was computed after registration of two T1w images (after INU-correction) using mri_robust_template^75^.

Brain surfaces were reconstructed using recon-all^76^, and the brain mask estimated previously was refined with a custom variation of the method to reconcile ANTs-derived and FreeSurfer-derived segmentations of the cortical gray matter of Mindboggle^77^. Volume-based spatial normalization to one standard space (MNI152NLin6Asym) was performed through nonlinear registration with antsRegistration (ANTs v.2.2.0), using brain-extracted versions of both T1w reference and the T1w template. The following template was selected for spatial normalization: FSL’s MNI ICBM 152 nonlinear 6th Generation Asymmetric Average Brain Stereotaxic Registration Model^78^ [RRID:SCR_002823; TemplateFlow ID: MNI152NLin6Asym].

#### Functional data preprocessing

For each of the seven BOLD runs per participant (across all tasks and sessions), the following preprocessing was performed. First, a reference volume and its skull-stripped version were generated using a custom methodology of fMRIPrep. A deformation field to correct for susceptibility distortions was estimated based on a field map that was coregistered to the BOLD reference, using a custom workflow of fMRIPrep derived from D. Greve’s epidewarp.fsl script and further improvements of HCP Pipelines^79^. Based on the estimated susceptibility distortion, an unwarped BOLD reference was calculated for a more accurate coregistration with the anatomical reference. The BOLD reference was then coregistered to the T1w reference using bbregister (FreeSurfer) which implements boundary-based registration^80^. Coregistration was configured with 9 d.f. to account for distortions remaining in the BOLD reference. Headmotion parameters with respect to the BOLD reference (transformation matrices, and six corresponding rotation and translation parameters) are estimated before any spatiotemporal filtering using mcflirt^81^.

BOLD runs were slice-time corrected using 3dTshift from AFNI 20190105 (ref. ^82^). The BOLD time-series (including slice-timing correction when applied) were resampled onto their original, native space by applying a single, composite transform to correct for headmotion and susceptibility distortions. These resampled BOLD time-series will be referred to as preprocessed BOLD in original space, or just preprocessed BOLD. The BOLD time-series were resampled into standard space, generating a preprocessed BOLD run in [‘MNI152NLin6Asym’] space. First, a reference volume and its skull-stripped version were generated using a custom methodology of fMRIPrep.

Additionally, several confounding time-series were calculated based on the preprocessed BOLD: framewise displacement (FD), DVARS and three regionwise global signals. FD and DVARS are calculated for each functional run, both using their implementations in Nipype^83^. The three global signals are extracted within the CSF, the WM and the whole-brain masks. Additionally, a set of physiological regressors were extracted to allow for component-based noise correction CompCor^84^. Principal components are estimated after high-pass filtering the preprocessed BOLD time-series (using a discrete cosine filter with 128 s cut-off) for the two CompCor variants: temporal (tCompCor) and anatomical (aCompCor). tCompCor components are then calculated from the top 5% variable voxels within a mask covering the subcortical regions. This subcortical mask is obtained by heavily eroding the brain mask, which ensures it does not include cortical GM regions. For aCompCor, components are calculated within the intersection of the aforementioned mask and the union of CSF and WM masks calculated in T1w space, after their projection to the native space of each functional run (using the inverse BOLD-to-T1w transformation). Components are also calculated separately within the WM and CSF masks. For each CompCor decomposition, the *k* components with the largest singular values are retained, such that the retained components’ time-series are sufficient to explain 50% of variance across the nuisance mask (CSF, WM, combined or temporal). The remaining components are dropped from consideration. The headmotion estimates calculated in the correction step were also placed within the corresponding confounds file. The confound time-series derived from headmotion estimates and global signals were expanded with the inclusion of temporal derivatives and quadratic terms for each^85^.

Frames that exceeded a threshold of 0.5 mm FD or 1.5 standardized DVARS were annotated as motion outliers. All resamplings can be performed with a single interpolation step by composing all the pertinent transformations (that is, headmotion transform matrices, susceptibility distortion correction when available and coregistrations to anatomical and output spaces). Gridded (volumetric) resamplings were performed using antsApplyTransforms (ANTs), configured with Lanczos interpolation to minimize the smoothing effects of other kernels^86^. Nongridded (surface) resamplings were performed using mri_vol2surf (FreeSurfer).

### fMRI data analysis

We implemented four event-related GLMs in SPM 12 to analyze the fMRI data. All GLMs included a button press regressor as a regressor of no interest. All regressors were convolved with a canonical haemodynamic response function. Because of the sensitivity of the blood oxygen level-dependent signal to motion and physiological noise, all GLMs included frame-wise displacement, six rigid-body motion parameters (three translations and three rotation), six anatomical component-based noise correction components (aCompCorr) and four cosine regressors estimated by fmriprep as confound regressors for denoising. Each block was modeled separately within the GLMs.

The first GLM modeled events during the picture-viewing task and contained separate onset regressors for each of the 12 stimuli. By modeling each stimulus separately, we could account for any stimulus-specific differences in activity driving the main effects and focus on distance-dependent modulations that ride on top of those stimulus-specific differences in activation. Each onset regressor was accompanied by two parametric regressors. These corresponded to the distance to the stimulus presented immediately before the current stimulus according to the spatial kernel and distance to the immediately preceding stimulus according to the predictive kernel. Both parametric regressors were z-scored, but not orthogonalized, so that any shared variance would be discarded. Trials where the same stimulus was repeated were modeled separately and stimuli immediately following a choice were excluded. Furthermore, the GLM contained an onset regressor for the choice trials. This was accompanied by two parametric regressors, reflecting chosen and an unchosen distance between the two stimuli and the preceding stimulus. Each of the three blocks on each day were modeled separately within the same GLM.

The second, third and fourth GLMs modeled events during the choice task. In these GLMs, three onset regressors were included, one indicating the choice period, the second indicating feedback times and the third corresponding to button presses. The duration of each event corresponded to the actual duration during the experiment. The choice period regressor was accompanied by two parametric modulators reflecting chosen and unchosen values of the stimuli as estimated by the winning model. Both were demeaned, but not orthogonalized.

In the second GLM, the feedback regressor was accompanied by a spatial weight updating signal. A trial-by-trial estimate of the influence of the spatial map on the choices was estimated, and the demeaned trial-by-trial difference was included as a parametric modulator.

In the third GLM, the feedback regressor was accompanied by a parametric regressor reflecting a prediction error signal computed based on the compositional map.

In the fourth GLM, the feedback regressor was accompanied by a parametric regressor reflecting the prediction error difference signal. Here, the reward prediction error was estimated separately for the spatial and the predictive map, and the demeaned difference between the absolute prediction errors was included as a parametric regressor.

The contrast images from the first level were smoothed spatially using a Gaussian kernel of 8 mm FWHM and images of all participants were then analyzed as a second-level random effects analysis. We report all our results in the hippocampal formation, as this was our a priori ROI, at an uncorrected cluster-defining threshold of *P* < 0.001, combined with peak-level FWE small-volume correction at *P* < 0.05. For the SVC procedure, we used a mask comprising hippocampus, entorhinal cortex and subiculum (Extended Data Fig. 6). Results in the striatum and orbitofrontal cortex are reported at a cluster-defining threshold of *P* < 0.001 uncorrected, combined with peak-level FWE small-volume correction at *P* < 0.05 within an orbitofrontal and a caudate mask (Extended Data Fig. 6). Activation in other brain regions was considered significant only at a level of *P* < 0.001 uncorrected if it survived whole-brain FWE correction at the cluster level (*P* < 0.05). While we used masks to correct for multiple comparisons in our ROIs, all statistical parametric maps presented in the manuscript are unmasked and thresholded at *P* < 0.01 for visualization.

To relate neural effects to behavioral parameters and to each other, we defined the following ROIs: spatial hippocampal map in session 3 from GLM 1 (Fig. 4a); hippocampal spatial weight update from GLM 2 (Fig. 5e); change in hippocampal map representation from session 2 to session 3 with hippocampal spatial weight update as covariate from GLM 1 (Fig. 5f); and OFC difference in relative map accuracy with hippocampal spatial weight update as covariate from GLM 3 (Fig. 5h). All voxels exceeding a threshold of *P* < 0.001 were included in an ROI if the cluster survived correction for multiple comparisons.

To estimate how much an effect covaried with behavioral effects, we included spatial and predictive weights, respectively (Fig. 4f), as well as the inference error (Fig. 4g) as a covariate on the second level and tested for significant effects. In Fig. 5f–h, we included the parameter estimate reflecting the size of the hippocampal spatial weight update signal (Fig. 5e) as a covariate.

### Statistics and reproducibility

All correlations used Pearson’s correlations and we report two-tailed *P* values. Data normality was assessed using the Lilliefors test. No statistical method was used to predetermine sample size, but the final sample size (*n* = 48) exceeds commonly accepted good practice in the field^6,8,22^. Data from four participants were excluded due to a scanner defect (*n* = 3) and problems during preprocessing (*n* = 1). The experiments were not randomized to different conditions. Data collection and analysis were not performed blind to the conditions of the experiments.

### Mediation analysis

We used the Mediation and Moderation Toolbox^41,42^ to perform two single-level mediation analyses (Fig. 4h and Fig. 5i). The total effect of the independent variable *X* on the dependent variable *Y* is referred to as path c. That effect is then partitioned into a combination of a direct effect of *X* on *Y* (path c′), and an indirect effect of *X* on Y that is transmitted through a mediator *M* (path ab). We also estimated the relationship between *X* and *M* (path a) as well as between *M* and *Y* (path b). This last path ‘b’ is controlled for *X*, such that paths ‘a’ and ‘b’ correspond to two separable processes contributing to *Y*. We determined two-tailed uncorrected *P* values from the bootstrap confidence intervals (CI) for the path coefficients^42^.

To test whether the spatial weights mediate the effect of hippocampal spatial map on the inference error, we defined *X* as each individual’s parameter estimate from the hippocampal ROI encoding the spatial map (ROI based on Fig. 4a). The mediator *M* corresponded to each participant’s spatial weight as estimated by the model fit to the choice data. The outcome variable *Y* was defined as a participant’s inference error.

To test for a significant mediation linking the OFC relative map accuracy signals (*X*) to the change in hippocampal spatial map (*Y*), we extracted parameter estimates from an orbitofrontal ROI tracking the evidence that an outcome is predicted by either of the two maps (*X*, ROI based on Fig. 5h) and related this to the change in spatial representation in the left hippocampus (*Y*, ROI based on Fig. 5f) via the spatial updating signal in the right hippocampus (*M*, ROI based on Fig. 5e).

### Reporting summary

Further information on research design is available in the Nature Portfolio Reporting Summary linked to this article.

## Online content

Any methods, additional references, Nature Portfolio reporting summaries, source data, extended data, supplementary information, acknowledgements, peer review information; details of author contributions and competing interests; and statements of data and code availability are available at 10.1038/s41593-023-01283-x.

## Supplementary information

Supplementary Information Supplementary Text.

Reporting Summary

## Data Availability

Raw behavioral data, unthresholded group-level statistical brain maps from neuroimaging analyses as well as source data to reproduce all figures are publicly available here: https://github.com/tankred-saanum/Cognitive-maps-for-rewards. Raw imaging data in BIDS format are publicly available on Openneuro: https://openneuro.org/datasets/ds004360 (ref. ^87^). Source data are provided with this paper.

## Source data

Source Data Fig. 2 Statistical source data.
Source Data Fig. 3 Statistical source data.
Source Data Fig. 4 Statistical source data.
Source Data Fig. 5 Statistical source data.

## Code availability

Task, analysis and computational modeling code are publicly available here: https://github.com/tankred-saanum/Cognitive-maps-for-rewards^88^.

## References

[1] Shepard RN (1987). Toward a universal law of generalization for psychological science. Science.

[2] Gershman SJ, Daw ND (2017). Reinforcement learning and episodic memory in humans and animals: an integrative framework. Annu. Rev. Psychol..

[3] Guttman N, Kalish HI (1956). Discriminability and stimulus generalization. J. Exp. Psychol..

[4] Kahnt T, Tobler PN (2016). Dopamine regulates stimulus generalization in the human hippocampus. eLife.

[5] Wu CM, Schulz E, Garvert MM, Meder B, Schuck NW (2020). Similarities and differences in spatial and non-spatial cognitive maps. PLoS Comput. Biol..

[6] Barron HC (2020). Neuronal computation underlying inferential reasoning in humans and mice. Cell.

[7] Brogden WJ (1939). Sensory pre-conditioning. J. Exp. Psychol..

[8] Baram AB, Muller TH, Nili H, Garvert MM, Behrens TEJ (2021). Entorhinal and ventromedial prefrontal cortices abstract and generalize the structure of reinforcement learning problems. Neuron.

[9] Wimmer GE, Daw ND, Shohamy D (2012). Generalization of value in reinforcement learning by humans. Eur. J. Neurosci..

[10] Morgan LK, MacEvoy SP, Aguirre GK, Epstein RA (2011). Distances between real-world locations are represented in the human hippocampus. J. Neurosci..

[11] O’Keefe, J. & Nadel, L. *The Hippocampus as a Cognitive Map* (Clarendon Press, 1978).

[12] Tolman EC (1948). Cognitive maps in rats and men. Psychol. Rev..

[13] Constantinescu AO, O’Reilly JX, Behrens TE (2016). Organizing conceptual knowledge in humans with a gridlike code. Science.

[14] Aronov D, Nevers R, Tank DW (2017). Mapping of a non-spatial dimension by the hippocampal-entorhinal circuit. Nature.

[15] Nau M, Navarro Schröder T, Bellmund JLS, Doeller CF (2018). Hexadirectional coding of visual space in human entorhinal cortex. Nat. Neurosci..

[16] Theves S, Fernández G, Doeller CF (2020). The hippocampus maps concept space, not feature space. J. Neurosci..

[17] Theves S, Fernandez G, Doeller CF (2019). The hippocampus encodes distances in multidimensional feature space. Curr. Biol..

[18] Deuker L, Bellmund J, Schröder TN, Doeller C (2016). An event map of memory space in the hippocampus.. eLife.

[19] Bellmund JLS, Polti I, Doeller CF (2020). Sequence memory in the hippocampal-entorhinal region. J. Cogn. Neurosci..

[20] Eichenbaum H (2014). Time cells in the hippocampus: a new dimension for mapping memories. Nat. Rev. Neurosci..

[21] Schapiro AC, Turk-Browne NB, Norman KA, Botvinick MM (2016). Statistical learning of temporal community structure in the hippocampus. Hippocampus.

[22] Garvert MM, Dolan RJ, Behrens TE (2017). A map of abstract relational knowledge in the human hippocampal–entorhinal cortex. eLife.

[23] Schuck NW, Cai MB, Wilson RC, Niv Y (2016). Human orbitofrontal cortex represents a cognitive map of state space. Neuron.

[24] Schapiro AC, Rogers TT, Cordova NI, Turk-Browne NB, Botvinick MM (2013). Neural representations of events arise from temporal community structure. Nat. Neurosci..

[25] Schapiro AC, Kustner LV, Turk-Browne NB (2021). Shaping of object representations in the human medial temporal lobe based on temporal regularities. Curr. Biol..

[26] Nieh EH (2021). Geometry of abstract learned knowledge in the hippocampus. Nature.

[27] Zheng, X. Y. et al. Parallel cognitive maps for short-term statistical and long-term semantic relationships in the hippocampal formation. Preprint at *bioRxiv*10.1101/2022.08.29.505742 (2022).

[28] Shahar N (2019). Credit assignment to state-independent task representations and its relationship with model-based decision making. Proc. Natl Acad. Sci. USA.

[29] Niv Y (2019). Learning task-state representations. Nat. Neurosci..

[30] Wikenheiser AM, Schoenbaum G (2016). Over the river, through the woods: cognitive maps in the hippocampus and orbitofrontal cortex. Nat. Rev. Neurosci..

[31] Schuck NW, Niv Y (2019). Sequential replay of nonspatial task states in the human hippocampus. Science.

[32] Wittkuhn L, Chien S, Hall-McMaster S, Schuck NW (2021). Replay in minds and machines. Neurosci. Biobehav. Rev..

[33] Stachenfeld KL, Botvinick MM, Gershman SJ (2017). The hippocampus as a predictive map. Nat. Neurosci..

[34] Saanum, T., Schulz, E. & Speekenbrink, M. Compositional generalization in multi-armed bandits. Preprint at https://psyarxiv.com/v6mzb/ (2021).

[35] Schulz E, Tenenbaum JB, Duvenaud D, Speekenbrink M, Gershman SJ (2017). Compositional inductive biases in function learning. Cogn. Psychol..

[36] Gershman SJ (2019). Uncertainty and exploration. Decision.

[37] Rigoux L, Stephan KE, Friston KJ, Daunizeau J (2014). Bayesian model selection for group studies-revisited. Neuroimage.

[38] Barron HC, Garvert MM, Behrens TE (2016). Repetition suppression: a means to index neural representations using bold?. Philos. Trans. R. Soc. Lond. B Biol. Sci..

[39] Grill-Spector K (2006). Selectivity of adaptation in single units: implications for fmri experiments. Neuron.

[40] Bellmund JLS, Deuker L, Montijn ND, Doeller CF (2022). Mnemonic construction and representation of temporal structure in the hippocampal formation. Nat. Commun..

[41] Wager TD, Davidson ML, Hughes BL, Lindquist MA, Ochsner KN (2008). Prefrontal-subcortical pathways mediating successful emotion regulation. Neuron.

[42] Atlas LY, Bolger N, Lindquist MA, Wager TD (2010). Brain mediators of predictive cue effects on perceived pain. J. Neurosci..

[43] Banerjee A (2020). Value-guided remapping of sensory cortex by lateral orbitofrontal cortex.. Nature.

[44] Takahashi YK (2011). Expectancy-related changes in firing of dopamine neurons depend on orbitofrontal cortex.. Nat. Neurosci..

[45] Schoenbaum G, Roesch MR, Stalnaker TA, Takahashi YK (2009). A new perspective on the role of the orbitofrontal cortex in adaptive behaviour. Nat. Rev. Neurosci..

[46] Howard LR (2014). The hippocampus and entorhinal cortex encode the path and Euclidean distances to goals during navigation. Curr. Biol..

[47] Chadwick MJ, Jolly AE, Amos DP, Hassabis D, Spiers HJ (2015). A goal direction signal in the human entorhinal/subicular region. Curr. Biol..

[48] Schuck, N. W., Wilson, R. & Niv, Y. in *Goal-Directed Decision Making* (eds Morris, R. et al.) Ch. 12 (Academic Press, 2018).

[49] Doeller CF, King JA, Burgess N (2008). Parallel striatal and hippocampal systems for landmarks and boundaries in spatial memory. Proc. Natl Acad. Sci. USA.

[50] Gallagher M, McMahan RW, Schoenbaum G (1999). Orbitofrontal cortex and representation of incentive value in associative learning. J. Neurosci..

[51] Wikenheiser AM, Marrero-Garcia Y, Schoenbaum G (2017). Suppression of ventral hippocampal output impairs integrated orbitofrontal encoding of task structure. Neuron.

[52] Boorman ED, Rajendran VG, O’Reilly JX, Behrens TE (2016). Two anatomically and computationally distinct learning signals predict changes to stimulus-outcome associations in hippocampus. Neuron.

[53] Zhou J (2021). Evolving schema representations in orbitofrontal ensembles during learning. Nature.

[54] Henson R, Shallice T, Dolan R (2000). Neuroimaging evidence for dissociable forms of repetition priming. Science.

[55] Müller NG, Strumpf H, Scholz M, Baier B, Melloni L (2012). Repetition suppression versus enhancement—it’s quantity that matters. Cereb. Cortex.

[56] Segaert K, Weber K, de Lange FP, Petersson KM, Hagoort P (2013). The suppression of repetition enhancement: a review of fMRI studies. Neuropsychologia.

[57] Wissig SC, Kohn A (2012). The influence of surround suppression on adaptation effects in primary visual cortex. J. Neurophysiol..

[58] Turk-Browne N, Yi D-J, Leber A, Chun M (2006). Visual quality determines the direction of neural repetition effects. Cereb. Cortex.

[59] Schlichting ML, Mumford JA, Preston AR (2015). Learning-related representational changes reveal dissociable integration and separation signatures in the hippocampus and prefrontal cortex. Nat. Commun..

[60] Favila SE, Chanales AJH, Kuhl BA (2016). Experience-dependent hippocampal pattern differentiation prevents interference during subsequent learning. Nat. Commun..

[61] Russek EM, Momennejad I, Botvinick MM, Gershman SJ, Daw ND (2017). Predictive representations can link model-based reinforcement learning to model-free mechanisms. PLoS Comput. Biol..

[62] Kondor, R. & Lafferty, J. D. (2002) Diffusion Kernels on Graphs and Other Discrete Structures. Proceedings of the International Conference on Machine Learning, 315–322.

[63] Schulz E, Franklin NT, Gershman SJ (2020). Finding structure in multi-armed bandits. Cogn. Psychol..

[64] Bates D, Mächler M, Bolker B, Walker S (2015). Fitting linear mixed-effects models using lme4. J. Stat. Softw..

[65] Stephan KE, Penny WD, Daunizeau J, Moran RJ, Friston KJ (2009). Bayesian model selection for group studies. Neuroimage.

[66] Daw ND, Gershman SJ, Seymour B, Dayan P, Dolan RJ (2011). Model-based influences on humans’ choices and striatal prediction errors. Neuron.

[67] Feinberg D (2010). Multiplexed echo planar imaging for sub-second whole brain fMRI and fast diffusion imaging. PLoS ONE.

[68] Moeller S (2010). Multiband multislice ge-epi at 7 tesla, with 16-fold acceleration using partial parallel imaging with application to high spatial and temporal whole-brain fmri. Magn. Reson. Med..

[69] Esteban O (2018). fMRIPrep: a robust preprocessing pipeline for functional MRI.. Nat. Methods.

[70] Gorgolewski K (2011). Nipype: a flexible, lightweight and extensible neuroimaging data processing framework in python. Front. Neuroinform..

[71] Gorgolewski, K. et al. (2011). Nipype: a flexible, lightweight and extensible neuroimaging data processing framework in Python. *Front. Neuroimform.* **5**, 13. 10.3389/fninf.2011.00013 PMC3159964 21897815

[72] Tustison NJ (2010). N4itk: improved n3 bias correction. IEEE Trans. Med. Imaging.

[73] Avants B, Epstein C, Grossman M, Gee J (2008). Symmetric diffeomorphic image registration with cross-correlation: evaluating automated labeling of elderly and neurodegenerative brain. Med. Image Anal..

[74] Zhang Y, Brady M, Smith S (2001). Segmentation of brain MR images through a hidden Markov random field model and the expectation-maximization algorithm. IEEE Trans. Med. Imaging.

[75] Reuter M, Rosas HD, Fischl B (2010). Highly accurate inverse consistent registration: a robust approach. NeuroImage.

[76] Dale AM, Fischl B, Sereno MI (1999). Cortical surface-based analysis: I. segmentation and surface reconstruction. NeuroImage.

[77] Klein A (2017). Mindboggling morphometry of human brains. PLoS Comput. Biol..

[78] Evans A, Janke A, Collins D, Baillet S (2012). Brain templates and atlases. NeuroImage.

[79] Glasser MF (2013). The minimal preprocessing pipelines for the human connectome project. NeuroImage.

[80] Greve DN, Fischl B (2009). Accurate and robust brain image alignment using boundary-based registration. NeuroImage.

[81] Jenkinson M, Bannister P, Brady M, Smith S (2002). Improved optimization for the robust and accurate linear registration and motion correction of brain images. NeuroImage.

[82] Cox RW, Hyde JS (1997). Software tools for analysis and visualization of fmri data. NMR Biomed..

[83] Power JD (2014). Methods to detect, characterize, and remove motion artifact in resting state fmri. NeuroImage.

[84] Behzadi Y, Restom K, Liau J, Liu TT (2007). A component based noise correction method (CompCor) for BOLD and perfusion based fmri. NeuroImage.

[85] Satterthwaite TD (2013). An improved framework for confound regression and filtering for control of motion artifact in the preprocessing of resting-state functional connectivity data. NeuroImage.

[86] Lanczos C (1964). Evaluation of noisy data. J. Soc. Ind. Appl. Math. Ser. B Numer. Anal..

[87] Garvert, M. M., Saanum, T., Schulz, E., Schuck, N. W. & Doeller, C. F. Cognitive maps for novel inference. 10.18112/openneuro.ds004360.v1.0.0 (2022).

[88] Saanum, T. & Garvert, M. tankred-saanum/cognitive-maps-for-rewards: release test v.01. *Zenodo*10.5281/zenodo.7486683 (2022).

