# Supplementary material for: Hippocampal spatio-predictive cognitive maps adaptively guide reward generalization
Source: Nat Neurosci. 2023 Apr 3;26(4):615–26. doi: 10.1038/s41593-023-01283-x (PMC10076220; doi:10.1038/s41593-023-01283-x)
Supplement: Supplementary file 1 — Supplementary Text. [file 41593_2023_1283_MOESM1_ESM.pdf]

nature neuroscience

Article

https://doi.org/10.1038/s41593-023-01283-x

Hippocampal spatio-predictive cognitive maps adaptively guide reward generalization

In the format provided by the authors and unedited

1 Deriving the predictive kernel

Given a participant's exploration run from day 1, we want a method for obtaining a transition matrix $\mathbf{T}(s,s')$, whose entries reflect the participant's propensity for venturing directly from stimuli s to stimuli s'. The successor representation (SR) is captured in the matrix $\mathbf{M}$, where entries $\mathbf{M}(s,s')$ equal the expected discounted number of future visits to stimulus s', starting from s. If we know the transition matrix $\mathbf{T}$ governing the one-step transition probabilities between every pair of stimuli, we can define the SR matrix $\mathbf{M}$ as the following infinite sum of $\mathbf{T}$ raised to the power of t

$$\mathbf{M} = \sum_{t=0}^{\infty} \gamma^t \mathbf{T}^t \tag{1}$$

where γ is the discount factor. This infinite sum can be computed analytically with matrix inversion

$$\mathbf{M} = (\mathbf{I} - \gamma\mathbf{T})^{-1} \tag{2}$$

where $\mathbf{I}$ is the identity matrix. Since we can compute the SR matrix $\mathbf{M}$ analytically from the transition matrix $\mathbf{T}$, we can attempt to recover the transition matrix from the SR matrix. This is fairly simple using matrix algebra. Since taking the matrix inverse of an inverted matrix gives us the uninverted matrix, $\mathbf{A}^{-1^{-1}} = \mathbf{A}$, we obtain

$$\mathbf{M}^{-1} = (\mathbf{I} - \gamma\mathbf{T}) \tag{3}$$

From Equation 3 we obtain $\mathbf{T}$ by subtracting the identity matrix $\mathbf{I}$, and dividing by $-\gamma$.

$$\mathbf{M}^{-1} - \mathbf{I} = -\gamma\mathbf{T} \tag{4}$$

$$\frac{\mathbf{M}^{-1} - \mathbf{I}}{-\gamma} = \mathbf{T} \tag{5}$$

leaving us with the transition matrix $\mathbf{T}$, which is such that performing an infinite random walk on it produces the SR matrix asymptotically.

2 Predictive relations explain reward generalization

We sought to verify that the particular exploration trajectory a participant took on day 1 actually influenced how that participant generalized about value, and that the predictive performance of the predictive and the spatio-predictive model could not be attributed to other, more general properties of the predictive kernels, for instance, that they are generally similar to the spatial kernel. To test this, we shuffled the assignments of the predictive kernels, so that each participant would have their choices predicted based on a kernel computed from an exploration trajectory they *themselves* had not taken. If participant choices and generalization were really driven by their specific interaction with the stimuli, then the predictive performance of a model based on the shuffled kernels should be substantially worse than the performance of a model using the correct exploration trajectories. We made the assignments symmetric (for a select pair of participant, their predictive kernels were swapped), and unique (no two participants could be assigned the same predictive kernel). As can be observed in Supplementary Figure 4e, there were several predictive kernels that were substantially correlated with each other. We reasoned that swapping correlated kernels would yield smaller differences in predictive performance. We therefore sought to generate our shuffled assignments so that the overall correlation would be as small as possible. To do this, we sampled new kernels for each participant based on their inverse correlation r^{-1} to the participant's true kernel. We sampled from a distribution obtained through a softmax transform

$$p_i(K_j) = \frac{exp(r_{ij}^{-1}/\tau)}{\sum_j^M exp(r_{ij}^{-1}/\tau)} \tag{6}$$

where K_j is the kernel of participant j, M is the number of participant minus participant i and those already assigned, and τ is the temperature parameter. τ plays a key role here, as it allows us to control the degree to which we sample exclusively from the least correlated kernels, as opposed to more uniformly from all other kernels. As τ increases, the distribution gets more uniform. We collected negative log-likelihoods from the predictive model predicting participant choices (Supplementary Figure 4f). We sampled 10 kernel assignments for 10 evenly spaced values for τ between 0.01 and 3, leaving us with 100 samples of

shuffled assignments, where the assignments were d with various degrees of uniformity. Substantiating the hypothesis that participant-specific predictive relations guide generalization, we observe that for all values of τ, the shuffled assignments (the red line) produce substantially worse fits (negative log-likelihood) to the choice data on average than the model using each participant's true predictive kernel (dashed blue line). Moreover, we observe that this loss is at its highest on average when we sample kernels more concentrated based on inverse correlations (lower τ), as opposed to more uniformly (higher τ) from the set of all kernels.

3 Hyper-parameters

The successor representation was learnt with temporal-difference learning, using a discount rate γ of 0.9. The signal variance parameter σ_f^2 of the Gaussian kernel (Equation 2) was set to 1, and the observation noise parameter σ^2 (Equation 1) was set to 0.01. The lengthscale parameter λ of the diffusion kernel (Equation 6) was set to 1. For the spatial model, the best-fitting lengthscale λ was 1.242. For the predictive model, the best-fitting learning rate η was 0.4125. For the spatio-predictive model, the best-fitting lenghtscale λ was 2.05, and the best-fitting learning rate η was 0.01. To create the kernel matrices used as predictors in the fMRI analyses, we used the spatial kernel with a lengthscale of 2.05 and the predictive kernel with a learning rate of 0.01, which gave the best fit for the spatio-predictive model. These best-fitting hyper-parameter configurations were used in modelling value ratings, and for the model recovery.

4 Model recovery analysis

We performed a model recovery analysis for our computational models, using their own best-fitting hyper-parameters. We first simulated choice behavior from our models based on the choices that the participants encountered in the experiment. As such, we obtained 4800 simulated decisions from our models. The predictive and spatio-predictive models used the predictive kernel of the participant at the corresponding trial. Choices were made deterministically to maximize expected reward, where the expected reward was estimated from previous observations. After each choice, the models received a reward which they used to condition predictions about rewards for subsequent trials. We then computed how likely each model was to produce the simulated choice behavior from all other models, including its own choice behavior. We were able to recover each model's behavior successfully: All models were by far the most likely to produce their own choice data.

5 Evidence-based map weighting

After each trial we computed the posterior probabilities of the spatial and predictive GP, given reward observations, $p(k \mid r) \propto p(r \mid k)p(k)$, with which we weighted the kernels for the compositional map. We implemented two versions of this model: One which updates the weights based on Bayes rule, and one which updates the weights towards the posterior by an amount α, reflecting the possibility that subjects may not integrate the evidence perfectly. We treat α as a hyperparameter and fit it to the population instead of the SR learning rate. We found that the model was more likely to generate choice data than the spatio-predictive model assuming equal weights, and considerably more likely than the model assuming that subjects updated how they used the maps according to a perfect Bayesian observer.

6 Symmetric predictive maps

Symmetric kernels are prerequisites for GP regression. However, participants may use *asymmetric* predictive relations to generalize. We tested whether asymmetric predictive relations explain generalization behaviour with a model which used the value function $\mathbf{V} = \mathbf{Mr}$ as a proxy for the reward function to make decisions, where $\mathbf{M}$ is the asymmetric successor matrix, and $\mathbf{r}$ the vector of monster rewards. This model did substantially worse than all GP-based models in predicting participant behavior. We thus maintain that Gaussian process regression using symmetrical kernels is the best model of participant behavior.
