# Supplementary material for: Hippocampal spatio-predictive cognitive maps adaptively guide reward generalization
Source: Nat Neurosci. 2023 Apr 3;26(4):615–26. doi: 10.1038/s41593-023-01283-x (PMC10076220; doi:10.1038/s41593-023-01283-x)
Supplement: Supplementary file 2 — Reporting Summary [file 41593_2023_1283_MOESM2_ESM.pdf]

nature portfolio

Corresponding author(s): Mona M. Garvert

Last updated by author(s): Dec 14, 2022

Reporting Summary

Nature Portfolio wishes to improve the reproducibility of the work that we publish. This form provides structure for consistency and transparency in reporting. For further information on Nature Portfolio policies, see our Editorial Policies and the Editorial Policy Checklist.

Statistics

For all statistical analyses, confirm that the following items are present in the figure legend, table legend, main text, or Methods section.

n/a	Confirmed	
☐	☒	The exact sample size (*n*) for each experimental group/condition, given as a discrete number and unit of measurement
☐	☒	A statement on whether measurements were taken from distinct samples or whether the same sample was measured repeatedly
☐	☒	The statistical test(s) used AND whether they are one- or two-sided<br>*Only common tests should be described solely by name; describe more complex techniques in the Methods section.*
☐	☒	A description of all covariates tested
☐	☒	A description of any assumptions or corrections, such as tests of normality and adjustment for multiple comparisons
☐	☒	A full description of the statistical parameters including central tendency (e.g. means) or other basic estimates (e.g. regression coefficient) AND variation (e.g. standard deviation) or associated estimates of uncertainty (e.g. confidence intervals)
☐	☒	For null hypothesis testing, the test statistic (e.g. *F*, *t*, *r*) with confidence intervals, effect sizes, degrees of freedom and *P* value noted<br>*Give P values as exact values whenever suitable.*
☒	☐	For Bayesian analysis, information on the choice of priors and Markov chain Monte Carlo settings
☒	☐	For hierarchical and complex designs, identification of the appropriate level for tests and full reporting of outcomes
☐	☒	Estimates of effect sizes (e.g. Cohen's *d*, Pearson's *r*), indicating how they were calculated

Our web collection on statistics for biologists contains articles on many of the points above.

Software and code

Policy information about availability of computer code

Data collection	The exploration and object location memory task were coded using the Python-based virtual reality software package Vizard (Version 4, Santa Barbara, CA: WorldViz LLC). MRI data were acquired using a 32-channel head coil on a 3 Tesla Siemens Magnetom SkyraFit system (Siemens, Erlangen, Germany). The tasks inside the MR scanner were presented on a rear-projection screen and implemented in Matlab R2016a using Psychtoolbox Toolbox version 3. Subsequently, outside of the scanner, participants performed a few short behavioral tasks in front of a computer screen, implemented with custom Matlab R2016a code.<br>fMRI scans were acquired using T2-weighted gradient-echo echo planar imaging (GE-EPI) with multiband acceleration.
Data analysis	Imaging and behavioral data analysis were carried out using fmriprep 1.4.0, SPM12 and Matlab R2021a. Computational models were developed using Python 3.6, R version 3.6.1 and Matlab2020b.

For manuscripts utilizing custom algorithms or software that are central to the research but not yet described in published literature, software must be made available to editors and reviewers. We strongly encourage code deposition in a community repository (e.g. GitHub). See the Nature Portfolio guidelines for submitting code & software for further information.

Data

Policy information about availability of data

All manuscripts must include a data availability statement. This statement should provide the following information, where applicable:

- Accession codes, unique identifiers, or web links for publicly available datasets
- A description of any restrictions on data availability
- For clinical datasets or third party data, please ensure that the statement adheres to our policy

Raw behavioural data, unthresholded group-level statistical brain maps from neuroimaging analyses as well as source data to reproduce all figures are publicly

available here: https://github.com/tankred-saanum/Cognitive-maps-for-rewards.
Raw imaging data in BIDS format is publicly available here:

Field-specific reporting

Please select the one below that is the best fit for your research. If you are not sure, read the appropriate sections before making your selection.

☒ Life sciences ☐ Behavioural & social sciences ☐ Ecological, evolutionary & environmental sciences

For a reference copy of the document with all sections, see nature.com/documents/nr-reporting-summary-flat.pdf

Life sciences study design

All studies must disclose on these points even when the disclosure is negative.

Sample size	52 data sets were collected, of those 48 participants were included in the final sample. No was analysis could be performed for this study since this is a new decision-making paradigm developed to test the influence of cognitive maps on choice that had never been tested before. The final sample size (n=48) exceeded the institute's common practices at the time of data acquisition.
Data exclusions	Due to a scanner defect, three participants could not be scanned on the third day and the data was excluded from the final sample. One participant was excluded due to technical problems during the preprocessing. For one participant, the final behavioral test could not be performed for technical reasons. No participant was excluded based on behavioral performance.
Replication	One fMRI study was conducted and group-level replication was not undertaken because of the resources needed for running this study (48 individuals tested over three subsequent days including 2 fMRI sessions). But the main effects are present in the majority of participants.
Randomization	Only one group of participants was tested and thus participants were not assigned to experimental groups.
Blinding	No group assignment took place, blinding was therefore not necessary.

Reporting for specific materials, systems and methods

We require information from authors about some types of materials, experimental systems and methods used in many studies. Here, indicate whether each material, system or method listed is relevant to your study. If you are not sure if a list item applies to your research, read the appropriate section before selecting a response.

Materials & experimental systems

n/a	Involved in the study	
☒	☐	Antibodies
☒	☐	Eukaryotic cell lines
☒	☐	Palaeontology and archaeology
☒	☐	Animals and other organisms
☐	☒	Human research participants
☒	☐	Clinical data
☒	☐	Dual use research of concern

Methods

n/a	Involved in the study	
☒	☐	ChIP-seq
☒	☐	Flow cytometry
☐	☒	MRI-based neuroimaging

Human research participants

Policy information about studies involving human research participants

Population characteristics	52 neurologically and psychiatrically healthy participants with normal or corrected-to-normal vision and suitability for undergoing MRI scanning took part in this study (age 26.8 ± 3.8 years, 20-34 years old, 27 male).
Recruitment	Participants were recruited using the participant database of the Max Planck Institute for Human Cognitive and Brain Sciences. Participants tended to be undergraduate or Master's students at the University of Leipzig and reflected the demographics of the local student population. We are not aware of any selection biases (self or others) that could have impacted the results.
Ethics oversight	The study was approved by the ethics committee at the Medical Faculty at the University of Leipzig (221/18-ek).

Note that full information on the approval of the study protocol must also be provided in the manuscript.

Magnetic resonance imaging

Experimental design

Design type	Task fMRI with event-related design
Design specifications	Participants performed three blocks of a picture-viewing task in the scanner on days 2 and 3, preceded by one block of the choice task on day 3. The picture viewing task consisted of 144 trials, where monsters were presented in a pseudo-random order. Each monster was presented for 2 seconds, with an inter-trial-interval drawn from a truncated exponential function with a mean of 3 seconds (between 2 to 5 seconds). Occasionally (on 24 trials), two monsters were presented simultaneously and participants had to indicate which of the two monsters was located closer in space or more similar in value to the monster they had seen immediately before. These trials were self-paced. Across participants, a block lasted in average 20:22 minutes (including self-paced trials).<br><br>In the choice task on day 3, participants were presented with two monsters until they indicated their selection by button press (self-paced). After an inter-trial interval, the outcome associated with the selection was presented for 2 seconds, followed by another inter-trial interval. Both intervals were again drawn from a truncated exponential function with a mean of 3 seconds (between 2 to 5 seconds). Participants performed 100 trials of the choice task.<br>Across participants, the choice task lasted in average 17:53 minutes.
Behavioral performance measures	In the scanner, we recorded button presses and response times on probe trials in the picture viewing task, as well as on all trials in the choice task. We assessed performance (percent correct) in the probe trial as well as in the choice task to test whether participants performed the task as expected.

Acquisition

Imaging type(s)	functional and structural MRI , fieldmap
Field strength	3T
Sequence & imaging parameters	MRI data were acquired using a 32-channel head coil on a 3 Tesla Siemens Magnetom SkyraFit system (Siemens, Erlangen, Germany). fMRI scans were acquired in axial orientation using T2*-weighted gradient-echo echo planar imaging (GE-EPI) with multiband acceleration, sensitive to blood oxygen level-dependent (BOLD) contrast.We collected 60 transverse slices of 2-mm thickness with an in-plane resolution of 2 x 2 mm, a multiband acceleration factor of 3, a repetition time of 2 s, and an echo time of 23.6 ms. Slices were tilted by 90 degrees relative to the rostro-caudal axis. Furthermore, a T1-weighted anatomical scan with 1 x 1 x 1 mm resolution was acquired. In addition, a whole-brain field map with dual echo-time images (TE1 = 5.92 ms, TE2 = 8.38 ms, resolution 2 x 2 x 2.26 mm) was obtained in order to measure and later correct for geometric distortions due to susceptibility-induced field inhomogeneities.
Area of acquisition	whole-brain

Diffusion MRI ☐ Used ☒ Not used

Preprocessing

Preprocessing software	Preprocessing was performed using fmriprep 1.4.0, which is based on Nipype 1.2.0. Preprocessing stages included brain extraction, correction for spatial distortion by applying the fieldmap, motion correction, slice-time correction, high-pass filtering. Contrast images were smoothed with a FWHM of 8 mm.
Normalization	2 T1-weighted (T1w) images were corrected for intensity non-uniformity (INU) with N4BiasFieldCorrection, distributed with ANTs 2.2.066. The T1w-reference was then skull-stripped with a Nipype implementation of the antsBrainExtraction.sh workflow (from ANTs), using OASIS30ANTs as target template. Brain tissue segmentation of cerebrospinal fluid (CSF), white-matter (WM) and gray-matter (GM) was performed on the brain-extracted T1w using fast. A T1w-reference map was computed after registration of 2 T1w images (after INU-correction) using mri_robust_template. Volume-based spatial normalization to one standard space (MNI152NLin6Asym) was then performed through nonlinear registration with antsRegistration (ANTs 2.2.0), using brain-extracted versions of both T1w reference and the T1w template.
Normalization template	FSL's MNI ICBM 152 non-linear 6th Generation Asymmetric Average Brain Stereotaxic Registration Model
Noise and artifact removal	A deformation field to correct for susceptibility distortions was estimated based on a field map that was co-registered to the BOLD reference image. Based on the estimated susceptibility distortion, an unwarped BOLD reference was calculated for a more accurate co-registration with the anatomical reference. BOLD runs were slice-time corrected using 3dTshift from AFNI. Head-motion parameters with respect to the BOLD reference (transformation matrices, and six corresponding rotation and translation parameters) were estimated before any spatio-predictive filtering using mcflirt.<br>All general linear models (GLMs) included, in addition to the regressors of interest, frame-wise displacement, six rigid-body motion parameters (three translations and three rotation), six anatomical component-based noise correction components (aCompCorr) and four cosine regressors estimated by fmriprep as confound regressors for denoising.
Volume censoring	The first five volumes were excluded from all analyses to allow for scanner calibration.

Statistical modeling & inference

Model type and settings	Univariate fMRI adaptation models. We first estimated contrasts for each subject as a first-level fixed-effects analysis and then combined data across subjects in a second-level random effects model.
Effect(s) tested	We implemented four types of event-related general linear models (GLMs) to investigate representational similarity using fMRI adaptation.<br><br>The first GLM modeled events during the picture viewing task and contained separate onset regressors for each of the twelve objects. These corresponded to the distance to the object presented immediately before the current object according to the spatial kernel and distance to the immediately preceding object according to the predictive kernel. Both parametric regressors were zscored, but not orthogonalized, so that any shared variance would be discarded. Trials where the same object was repeated were modeled separately and objects immediately following a choice were excluded. Furthermore, the GLM contained an onset regressor for the choice trials. This was accompanied by two parametric regressors, reflecting chosen and an unchosen distance between the two objects and the preceding object. Each of the three blocks on each day were modeled separately within the same GLM.<br>A seperate GLM was set up for each session (day 2 and day 3). We tested for a parametric modulation by including spatial and predictive relations on day 3 (after the choice task), as well as for a change in those effects from day 2 to day 3 by computing the difference between the two contrast images.<br><br>The second, third and fourth GLM modeled events during the choice task on day 3. In both GLMs, three onset regressors were included, one indicating the choice period, the second one indicating feedback times and the third one corresponding to button presses. The choice period regressor was accompanied by two parametric modulators reflecting chosen and unchosen values of the objects as estimated by the winning model. Both were demeaned, but not orthogonalized.<br>In the second GLM, the feedback regressor was accompanied by a spatial weight updating signal. A trial-by-trial estimate of the influence of the spatial map on the choices was estimated, and the demeaned trial-by-trial difference was included as a parametric modulator.<br><br>In the third GLM, the feedback regressor was accompanied by a parametric regressor reflecting a prediction error signal computed based on the compositional map.<br><br>In the fourth GLM, the feedback regressor was accompanied by a parametric regressor reflecting the prediction error difference signal. Here, the reward prediction error was estimated separately for the spatial and the predictive map, and the demeaned difference between the absolute prediction errors was included as a parametric regressor.

Specify type of analysis: ☐ Whole brain ☐ ROI-based ☒ Both

Anatomical location(s)	ROIs were defined functionally in an unbiased way based on an orthogonal contrast.<br>Masks for small-volume correction were defined anatomically, and included bilateral hippocampus, entorhinal cortex and subiculum.
Statistic type for inference (See Eklund et al. 2016)	Whole-brain analyses were performed voxel-wise. ROI analyses were performed on mean parameter estimates averaged across voxels within a given region of interest.
Correction	We performed family-wise error correction.<br>Effects in the hippocampal formation (our a priori ROI) are reported at a cluster-defining threshold of $p < 0.001$, combined with peak-level family-wise error (FWE) small-volume correction at $p < 0.05$ within an anatomically defined ROI. Results in the orbitofrontal cortex and striatum are reported at a cluster-defining threshold of $p < 0.001$ uncorrected, combined with anatomically defined orbitofrontal cortex and caudate masks, respectively.

Models & analysis

n/a	Involved in the study
☒	☐ Functional and/or effective connectivity
☒	☐ Graph analysis
☒	☐ Multivariate modeling or predictive analysis
